# Transcription Factors Associated with Leaf Senescence in Crops

**DOI:** 10.3390/plants8100411

**Published:** 2019-10-14

**Authors:** Sofia Bengoa Luoni, Francisco H. Astigueta, Salvador Nicosia, Sebastian Moschen, Paula Fernandez, Ruth Heinz

**Affiliations:** 1 Consejo Nacional de Investigaciones Científicas y Técnicas, Ciudad Autónoma de Buenos Aires 1425, Argentina; bengoaluoni@gmail.com (S.B.L.); ASTIFRAN10@GMAIL.COM (F.H.A.); NICOSIA.SALVADOR@INTA.GOB.AR (S.N.); MOSCHEN.SEBASTIAN@INTA.GOB.AR (S.M.); HEINZ.RUTH@INTA.GOB.AR (R.H.); 2 Escuela de Ciencia y Tecnología, Universidad Nacional de San Martín, San Martín, Buenos Aires 1650, Argentina; 3 Universidad Nacional de Lujan, Cruce Rutas Nac. 5 y 7, Lujan, Buenos Aires 6700, Argentina; 4 Instituto Nacional de Tecnología Agropecuaria, Estación Experimental Agropecuaria Famaillá, Tucumán 4142, Argentina; 5 Instituto de Agrobiotecnología y Biología Molecular (INTA-CONICET), Instituto de Biotecnología, Centro de Investigaciones en Ciencias Agronómicas y Veterinarias, Instituto Nacional de Tecnología Agropecuaria, Hurlingham, Buenos Aires 1686, Argentina; 6 Facultad de Ciencias Exactas y Naturales, Universidad de Buenos Aires, Ciudad Autónoma de Buenos Aires 1428, Argentina

**Keywords:** leaf senescence, crops, transcription factors, stay-green, yield

## Abstract

Leaf senescence is a complex mechanism controlled by multiple genetic and environmental variables. Different crops present a delay in leaf senescence with an important impact on grain yield trough the maintenance of the photosynthetic leaf area during the reproductive stage. Additionally, because of the temporal gap between the onset and phenotypic detection of the senescence process, candidate genes are key tools to enable the early detection of this process. In this sense and given the importance of some transcription factors as hub genes in senescence pathways, we present a comprehensive review on senescence-associated transcription factors, in model plant species and in agronomic relevant crops. This review will contribute to the knowledge of leaf senescence process in crops, thus providing a valuable tool to assist molecular crop breeding.

## 1. Introduction

Senescence is a natural phenomenon highlighted by a reduction in leaf functionality, clearly identified by changes in leaf color. It is a controlled process in which functional structures from leaves are dismantled to generate nutrients that are recycled and remobilized into developing organs, like young leaves, flowers and grains [1,2,3]. Because of climate change, plants have developed various strategies to respond efficiently to the changing environment. Under optimal conditions, the onset of leaf senescence depends mainly on the ontogeny of the plant. This process, however, can be induced prematurely by endogenous and exogenous stimuli, like biotic or abiotic stress conditions, to accelerate the remobilization of nutrients [1]. In annual species, this process provides enough energy to start the reproductive stage, in order to culminate its life cycle and generate offspring. In perennial species, on the other hand, recycling of nutrients implies the initiation of the vegetative rest stage [4].

Chloroplasts are the first organelles that begin to lose functionality. Their structures are dismantled by specific enzymes and chlorophyll is massively degraded, thus becoming a readily accessible source of nitrogen and causing leaf yellowing [5]. Free amino acids are remobilized or used as an alternative energy source during mitochondrial respiration [6]. Meanwhile, macromolecules, like fatty-acids and nucleic acids, are degraded and the nutrients derived from this catabolism are dynamically transported to younger tissues [7]. Micronutrients like Fe, Cu, Mn and Zinc are essential for seed germination and have an important role in grain quality. Nutrient remobilization of senescent leaves and root uptake occur mainly through the phloem [8,9]. However, the ions remobilization can vary between species and because of growth conditions [10,11]. On the other hand, leaf cells have different mechanisms regarding nutrient recycling, for example, they can degrade pigments inside the chloroplast [12], or stomal proteins inside the central vacuole or via senescence-associated vacuoles (SAV) [13,14].

During leaf development, phytohormones and other biochemical compounds regulate different signaling pathways mainly by modulating the activation or repression of different genes including transcription factors (TFs) [15]. Studies on leaf senescence, along with the methods used to analyze and describe this process, especially at the molecular level, have increased over the last years [2,16,17,18,19,20,21]. Next generation sequencing (NGS) technologies have increased our knowledge on this process [22,23,24,25]. TFs are involved in different developmental processes and many of them act as putative master regulators and hub genes in signaling processes [1,16,25,26,27]. Multiple studies have described correlations between transcription expression patterns and biological functions of TFs, mainly to elucidate the molecular mechanism between leaf senescence, photosynthesis and nutrient remobilization [28].

In this review, we describe the main regulating networks governing senescence in *Arabidopsis* and present evidence regarding how TFs, particularly from the NAC (NAM, ATAF and CUC) transcription factor family, interact with phytohormones and regulate the onset of leaf senescence. Moreover, we discuss how the modulation of TFs may contribute to the generation of delay-senescence varieties, named as “stay-green”, in important crops. The presented evidence confirms the importance of these genes as candidate tools to assist genetic breeding programs. Finally, we describe stay-green phenotype examples as a result of plant breeding tools, not only for grain-filling, but also for species in which greenery or post-harvest leaf lifespan needs to be improved, mainly in horticultural and ornamental industries. Hence, this work presents valuable information for the generation of biotechnological tools to assist molecular crop improvement.

## 2. Senescence Regulation Network in *Arabidopsis* and NAC TF Contribution

Advances in high-throughput technologies applied to gene expression studies triggered the identification of multiple senescence-associated genes (SAGs). In *Arabidopsis*, almost 20% of the genes change their expression during natural senescence [29]. These genes participate in different molecular, biochemical, morphological and physiological events that contribute to the senescence phenotype. Under stress conditions, plants rapidly adjust their physiology through the biosynthesis of phytohormones that promote stress-resistance responses or premature senescence. Phytohormones like ethylene, abscisic acid, jasmonates, auxins and salicylic acid promote senescence, whereas cytokinins and gibberellin acid delay this process.

TFs are nodes in gene expression pathways and a single TF can modulate an entire response process [26]. Thereby, the identification of senescence-associated TFs acting downstream of a hormone-signaling network could have an important impact on generating new tools for crop breeding. Different TF families participate in leaf senescence in many species, particularly the NAC [17,20,30,31,32,33], MYB [34,35], AP2 [36,37] and WRKY [38,39,40,41] families. The crosstalk regulation between phytohormones and TFs associated with senescence is crucial to understand the molecular mechanisms governing this last developmental stage.

**Figure 1:**
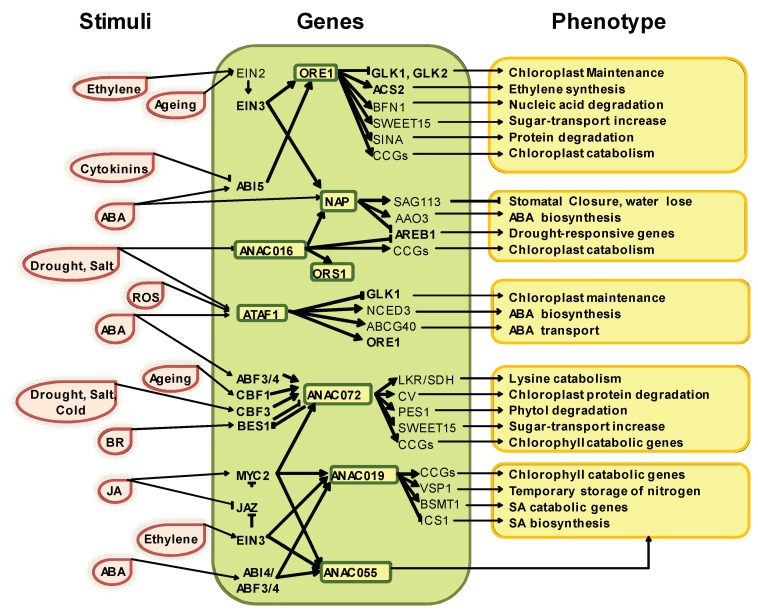
NAC (NAM, ATAF and CUC) TF (transcription factor) senescence network in *Arabidopsis*. Straight arrows or lines stand for direct interactions either protein–protein or protein–DNA, whereas light arrows represent functional relationships in the regulation network. Arrowheads represent positive (→) or negative (┤) regulation.

The NAC (NAM, ATAF and CUC) gene family is one of the largest groups of plant TFs, with more than 100 members in *Arabidopsis* [42]. The NAC proteins contain a NAC domain (InterPro IPR003441) at the N-terminal region, which is subdivided in five well-conserved subdomains (A–E), and very variable transcription regulatory regions (TRRs) at the C-terminal region [42]. The NAC domain is involved in dimerization and DNA binding, whereas the TRR region acts as a transcription activator or repressor [42]. According to global transcriptome profiling data, more than 30 NAC genes have enhanced expression during natural leaf senescence in *Arabidopsis* and maintain a strong crosstalk with phytohormones and environmental signals, which highlights their importance in the regulation of this process (Figure 1) [15]. ORE1, NAP, ANAC16, ATAF1, ANAC072, ANAC019 and ANAC055 promote leaf senescence, whereas VNI2 and JUB1 delay it [30,31,33,43,44,45,46].

Ethylene is a key senescence-promoting hormone. Indeed, its exogenous application accelerates leaf and flower senescence, whereas inhibitors of ethylene perception or biosynthesis delay leaf senescence [47]. Besides, senescing tissues display an elevated expression of genes encoding ethylene biosynthetic enzymes [26,48]. Several studies have suggested that ethylene response depends mainly on leaf ontogeny (or “natural” senescence), instead of stress-induced senescence [49,50,51]. Hence, in *Arabidopsis* senescence cannot be induced by ethylene until a defined developmental stage [49,51]. The ethylene response includes the activation of the EIN2 (ETHYLENE-INSENSITIVE 2) transcription factor, as evidenced by an *ein2* mutation that produced a direct impact on senescence phenotype [24,52]. Furthermore, EIN3 (ETHYLENE-INSENSITIVE 3), downstream EIN2, directly regulates chlorophyll catabolic genes *(CCG)*, like *NYE* (STAYGREEN), *NYC1* (NONYELLOW COLORING1) and *PAO* (PHEOPHORBIDE A OXYGENASE) [18].

ORE1 is one of the most studied TFs in leaf senescence. This TF is expressed during ethylene-induced senescence under the control of EIN2 [17]. In addition, ORE1 interacts with GOLDEN2-LIKE1 (GLK1) and GLK2, which are relevant for chloroplast development and maintenance [53]. Hetero-dimerization of ORE1 and GLKs inhibits the transcriptional activity of GLKs, leading to leaf senescence [53]. Furthermore, ORE1 accelerates chlorophyll lost by directly activating the transcription of Chlorophyll Catabolic Genes (*CCGs)* [18], as well as *BFN1* (BIFUNCTIONA NUCLEASE 1), *SAG29/SWEET15* (SENESCENCE-ASSOCIATED GENE 29/SUGARS WILL EVENTUALLY BE EXPORTER TRANSPORTERS 15) and *SINA* (SEVEN IN ABSENTIA) [20]. Consequently, ORE1 induces distinctive senescence features, like the degradation of nucleic acid, proteins and nitrogen recycling as well as the promotion of sugar transport [20,53]. Moreover, in *Arabidopsis* leaves, ORE1 promotes ethylene biosynthesis by positive feedback, through the transcriptional activation of ACS2 (ETHYLENE BIOSYNTHESIS GENE) [18].

The transcript level of *NAP* (NAC-LIKE ACTIVATED BY AP3/PI), another positive regulator of senescence, increases with leaf age [31]. In *Arabidopsis*, *ore1* and *nap* mutants revealed that these genes act in distinct and overlapping signaling pathways [24]. Phenotypic reversion in mutant plants further demonstrated that ORE1 and NAP can activate common as well as different NAC TF genes. In addition, EIN3 directly binds and upregulates both *NAP* and *ORE1* expression in ethylene-induced senescence [24].

Moreover, some studies have revealed that the regulating networks governing leaf senescence maintain a crosstalk between activating stimuli from different phytohormones making this process even more complex [54]. For example, EIN2 is also upregulated by abscisic acid (ABA) or salt [55,56,57,58].

ABA is an important signaling molecule that enables plants to tolerate unfavorable environmental stresses like drought, salt, cold, heat and oxidation [59,60]. Moreover, ABA can induce the expression of *CCGs* [61]. Two main families of ABA TFs were associated with senescence, ABA INSENSITIVE 3/4/5 (ABI3/4/5) and ABA-responsive element binding factor members (ABF1, AREB1/ABF2, AREB2/ABF4 and ABF3) [60].

ABI5 plays a crucial role during senescence by binding to the promoter of *ORE1* [62] and therefore activating *CCG* genes [63]. Likewise, NAP is a positive regulator of ABA concentrations inside the cell by binding the promoter of *AAO3* (ABSCISIC ALDEHYDE OXYDASE3) [64]. AAO3 is responsible for the final step in ABA biosynthesis [65]. Zhang and Gan [66] reported a mechanism that includes ABA–NAP–SAG113 signaling and demonstrated that *NAP* expression increases in an ABA-dependent manner. NAP upregulates the *SAG113* gene (SENESCENCE-ASSOCIATED GENE113), which in turn regulates stomatal movement and water loss in senescent leaves [66]. Conversely, cytokinin would efficiently promote the proteasomal degradation of ABI5 and, in this way, cytokinin may delay senescence by antagonizing the ABA effect [67]. Therefore, the balance of ABA and cytokinin concentration could be controlling the onset of age-induced senescence during plant development [68].

NAC016 is a positive regulator of senescence and can upregulate *NAP* and *CCGs* by direct binding to the gene promoter [43,63,69]. *NAC016* is also upregulated under abiotic stress and seems to downregulate ABA-dependent genes, presumably through AREB1 [63,70,71]. Yeast one-hybrid assays, on the other hand, suggest ORS1 as a direct target of NAC016 [43]. *nac16* and overexpressing *NAC016* plants showed contrasting expression patterns for *ORS1* [43]. ORS1 is a positive regulator of senescence in *Arabidopsis* and, according to a sequence analysis, a paralog to ORE1 [30]. Overexpression of *ORS1* produced an early senescence phenotype along with the upregulation of several SAGs. In addition, ORS1 is associated with senescence triggered by salinity and peroxide stress [30]. ATAF1, a positive senescence-regulated NAC TF, was upregulated by ABA, reactive oxygen species (ROS) treatment and drought stress [44,57]. ATAF1 possibly induces ABA biosynthesis by interacting with the *NCEDs* (9-cis-epoxycarotenoid dioxygenases) promoter, which is a key regulatory step of ABA biosynthesis [72]. ATAF1 also interferes in chloroplast maintenance by blocking *GLK1* transcription and inducing *ORE1* expression [44].

Additionally, Hickman et al. [73] reported *ANAC019*, *ANAC055* and *ANAC072 (RD26)* as *SAGs*. The study of these three genes is a difficult task because of their sequence similarity, leading to overlapping functions in downstream networks [74]. Single mutants of any of these three genes does not lead to detectable changes in phenotype, thus multiple mutants in the same plant were necessary for that purpose [75,76,77,78,79]. Evidence suggests that these three genes are induced under salt and drought stress [73,80,81], whereas only *NAC072* is induced also by cold treatment [73,80]. Moreover, the three NAC TFs are ABA-inducible genes [73,76,80,81,82]. ABF3 and ABF4 can directly bind the promoters of *ANAC072*, *ANAC055* and *ANAC019*, and activate their transcription [73,82]. The TF family comprised by CBF1 (*C-REPEAT BINDING FACTOR*), 2, 3 and 4 are important for regulating responses to abiotic stress. Particularly CBF3 is associated with abiotic stress, like cold, osmotic and salinity, whereas CBF1 is associated with ageing senescence. Interestingly, both genes can upregulate *ANAC072* [73,80].

ABA and brassinosteroids (BRs) antagonistically regulate senescence. BRs regulate plant growth and stress responses through the BES1/BZR1 (BRI1-EMS-SUPPRESSOR1) transcription factor family [83]. NAC072 is associated with the crosstalk between ABA and BR responses. Several studies show that BES1 is capable of inhibiting *NAC072* transcription [73,84,85]. Besides, Ye et al. [85] have demonstrated that NAC072 can downregulate BR gene responses (negative crosstalk) by generating a complex with BES1. During ABA-response stress, the ratio between ABA/BR increases and *ANC072* is upregulated thus triggering senescence. Furthermore, studies using the triple mutant *anac072*, *anac055* and *anac019* suggested a redundant function in the inhibition of BR-response genes [85]. As in the case of the balance between ABA/Cytokinins controlling the activation of *ABI5* described above, the regulation of *ANAC072* by ABA/BR is another example of control of senescence onset by phytohormone response genes.

Jasmonate (JA) is another phytohormone capable of inducing leaf senescence through the activation of *CCGs* in *Arabidopsis* [77,86]. Additionally, senescence can induce jasmonate biosynthesis [86,87]. The metabolic network is negatively regulated by the JAZ (JASMONATE ZIM-DOMAIN) transcription factors and activated by MYC2 [88]. Abundant evidence suggests that ANAC055, ANAC072 and ANAC019 act downstream of MYC2 during senescence [75,77,78,81,89]. Moreover, the protein MYC2 directly interacts with ANAC019 and in turn the protein complex activates *CCGs* [77].

ANAC055 and ANAC019 also have an important role in ethylene senescence response [24,52,75]. For instance, *ein2* plants displayed a considerable decrease in *ANAC055* and *ANAC019* expression, thus indicating an EIN2-dependent regulation. Indeed, EIN3 can bind *ANAC055* and *ANAC019* promoters [54]. Moreover, a crosstalk between ethylene and JA networks could explain the interaction of MYC2 and *ANAC019* during ethylene responses [54,90,91]. By contrast, *ANAC072* expression remains unaltered in *ein2* plants, which suggests that these two NAC members trigger senescence through different pathways [24].

ANAC072 can directly activate the transcriptional expression of *CCGs* [92]. Besides, ANAC072 is implicated in the metabolic reprogramming during senescence by controlling the expression of multiple genes across the cellular degradation hierarchy [93]. For example, ANAC072 activates the transcription of *CV* (CHLOROPLAST VESICULATION), which encodes a protein crucial for chloroplast protein degradation, and the sugar transport gene *SWEET 15* [93]. Besides, ANAC072 activates the transcription of genes involved in the catabolism of lysine and phytol. ANAC072 also reduces GABA (γ-aminobutyric acid) concentration by inducting the respective catabolic genes. This degradation, along with the degradation of lysine and phytol, provides substrates for cellular respiration during senescence [93].

ANAC019 can induce the expression of *CCGs*, but also interacts with the *VSP1* (VEGETATIVE STORAGE PROTEIN 1) promoter [75]. In turn, VSP1, an important source of mobilized nutrients, is transcriptionally activated by JA and wounding [94]. On the other hand, ANAC019 directly interacts with the promoters *ICS1* (ISOCHORISMATE SYNTHASE 1) and BSMT1 (S-ADENOSYLMETHIONINE-DEPENDENT METHYL-TRANSFERASE). ICS1 is involved in SA biosynthesis, whereas BSMT1 is associated with SA metabolism. ANAC019 represses *ICS1* and activates *BSMT1*, which leads to a reduction of SA accumulation [78]. To date, there is no substantial evidence of ANAC055 directly downregulating any gene. However, since ANAC019 and ANAC055 bind to the same DNA elements [81], ANAC055 may also bind to *BSMT1* and *ICS1* promoters and therefore regulate their expression [78].

NAC family genes can regulate senescence in a positive or negative manner. ANAC017, ANAC090 and ANAC082, named as “NAC troika” by Kimet et al. [95], were upregulated during the pre-senescence stage. These genes are negative regulators of senescence, their expression is highly linked to SA and ROS stress pathways. Besides, mutant plants for these genes showed accelerated senescence, whereas overexpression of these genes had the opposite effect. These genes are also involved in the downregulation of other NACs, suggesting that troika genes may underlie the positive-to-negative regulatory shift in senescence onset [95].

VNI2 is another negative regulator of senescence whose expression increases along with leaf aging and senescence [46]. Interestingly, *VNI2* expression accompanies leaf aging and senescence. Moreover, *vni2* plants showed an accelerated senescence, whereas its overexpression displayed a delayed senescence phenotype. Additionally, *VNI2* is upregulated with high salinity in an ABA-dependent manner [46]. Based on these findings, Seo et al. [96] proposed a model where the overregulation of *VNI2* can enhance stress resistance to ensure reproductive success. Similarly, Wu et al. [45] reported another NAC repressor of leaf senescence: JUNGBRUNNEN1 (JUB1). *JUB1* overexpression strongly delays senescence in *Arabidopsis*. Its transcription is activated by elevated hydrogen peroxide levels and enhances tolerance to various abiotic stresses. Moreover, *jub1* knockdown plants showed an early senescence phenotype [45].

## 3. The Stay-Green Trait in Crops

**Figure 2:**
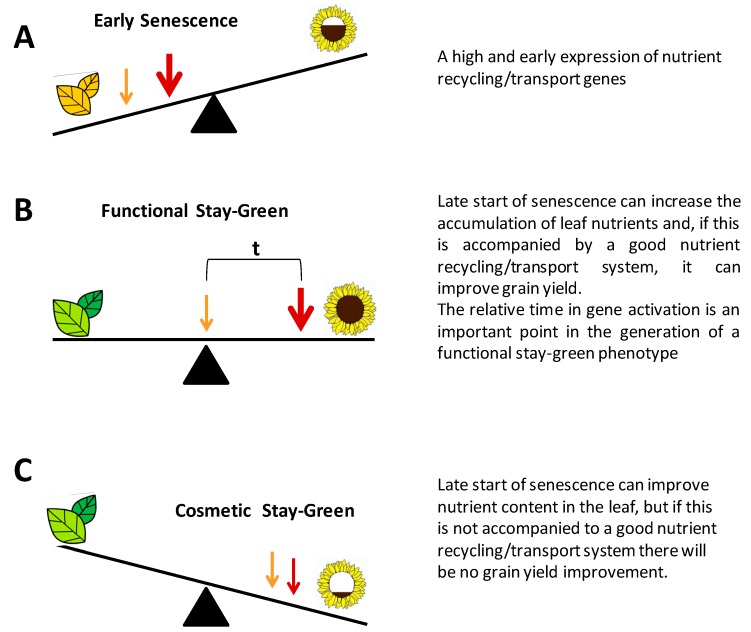
Stay-green phenotype vs. recycling/transport nutrient process. Orange arrows represent upregulation of genes that contribute to the onset of the senescence process, whereas red arrows represent upregulation of genes related to the catabolic leaf process and nutrient transport. (**A**), (**B**) and (**C**) correspond to Early Senescence, Functional Stay-Green and Cosmetic Stay-Green, respectively.

Along the senescence process, upregulation of the *CCGs*, *SWEET15*, *PES1*, *CV*, *SINA* and other genes are essential for leaf catabolic processes. The importance of these genes relies on that their capacity to increase the nutrient recycling and transport to the sink and, therefore, they are fundamental for the quality and yield of grains. The control of the expression of these genes may act as a breeding tool for the improvement in the grain yield. Interestingly, Uauy et al. [97] described a wheat line with an early senescence phenotype and a high grain quality, possibly because of an efficient catabolic process (this case is described below). A premature upregulation of these genes, however, can generate an early senescence phenotype and therefore a decline in grain quality, because of the shortage of nutrients in young leaves (Figure 2A).

On the other hand, stay-green varieties are plants that present a delay in foliar senescence while maintaining their green leaf area active for longer periods. The stay-green phenotype is an indicative of plant health in fields and can be associated with an increased tolerance to diseases, pests and drought [98]. For instance, in some species, the stay-green phenotype has been associated with diminished percentage of plant lodging [98]. Many researchers have suggested that the delay of leaf senescence may have a positive impact on agronomic yields, presumably by maintaining the photosynthetic machinery active, despite of the adverse condition especially during the reproductive stage [19,20,99]. In line with this finding, many studies have reported positive correlations between green leaf area, late senescence and productivity (e.g., in maize, sorghum, wheat, sunflower, rice and soybean). Moreover, in all these cases, this late senescence led to higher crop yields [38,100,101,102,103,104,105,106,107,108,109,110]. However, this positive effect depends on the interaction of each species with the environment. Furthermore, once senescence is triggered, the recycling events must occur efficiently and need to synchronize with leaf age to maximize the nutrient content (Figure 2B). However, if the stay-green phenotype is not accompanied by the catabolic process, no improvement occurs in the grain yield. This feature is known as cosmetic stay-greens (Figure 2C).

The grain-filling period is one of the most crucial stages in agronomic crops and depends on the relationship between the source and the sink. This relationship could be modified by an improved stay-green breeding program, i.e., the use of a stronger source may prolong the activity of the photosynthetic machinery. Different molecular tools have been widely used to study and achieve stay-green phenotypes in multiple species. In general, wild-type genotypes and plant varieties have provided an abundant source of stay-green germplasm. Moreover, these genotypes are useful for the detection of QTL and genes involved in senescence.

Alternatively, these genotypes can be used to study the transcriptional expression patterns and the biological function of the assessed genes. Several genes, defined as SAGs (senescence-associated genes), vary their expression throughout senescence. Among these genes, the transcription factors are particularly important candidates in the generation of functional stay-green crops [1]. Therefore, functional analyses are essential to confirm the real influence of these genes in senescence. Researchers usually assess, and often confirm, the role of candidate genes by overexpressing or silencing the candidate genes.

## 4. Main TFs Associated with Leaf Senescence in Crops

A leaf senescence delay has a strong impact on yield in several crops. Main relevant contributions were performed in *Arabidopsis*, and later on assessed in monocotyledonous plants like rice, wheat, barley, maize and sorghum, or in dicotyledonous crops like sunflower, soybean, cotton and grapevine [28,110,111,112]. Below, we describe the TF associated to senescence in conventional and non-conventional agronomic crops, with special attention on NAC TFs.

### 4.1. Rice

Rice (*Oryza sativa*) is one of the most important crops worldwide and provides the essential caloric requirement for more than half of the global population [113]. However, rice production will need to be increased by 40% in 2030 to satisfy food demands [114]. A few NAC TFs in rice were associated with leaf senescence and therefore are candidates for future agronomic improvement in rice breeding.

Several studies have linked the expression of *OsNAP*, a NAP-like transcription factor (Os03g21060), to the onset of leaf senescence in an age-dependent manner, as well as in JA and ABA treatments [115,116,117]. Plants overexpressing this gene had an early senescence phenotype and an elevated transcription of JA signaling genes (*LOX2* and *AOC1*). Moreover, knockdown plants showed a delay in senescence and downregulation of JA genes (*AOS*, *AOC1*, *OPR7*) [117]. *osnap* plants showed reduced ABA content, whereas *aba1* and *aba2* plants, two knock-out ABA biosynthesis gene plants, showed repressed expression of *OsNAP* [116]. Further analyses determined that OsNAP can directly bind to the promoter of genes related to chlorophyll degradation [116]. As expected, because of its homology with AtNAP, OsNAP also plays a crucial role in regulating senescence; in fact, it was also implicated in abiotic stress responses like high salinity, drought and low temperature [115]. Additionally, two independent studies demonstrated that OsNAP-repressed transgenic lines have a functional stay-green phenotype. Firstly, Liang et al. reported two independent knock-down plants showing an increase in grain yield of 6.3% and 10.3%, respectively [116]. Similarly, Tang et al. [118] confirmed that OsNAP acts as a functional stay-green. They reported that transgenic OsNAP-repressed rice plants increased the number of grains per plant (11%) as well as the grain weight (10%) and spikelet fertility rate (6%). Overall, these transgenic lines increased the grain yield per plant approximately 24% in comparison with the wild type.

Regarding OsNAC002 (Os04g0460600, ortholog of AtORE1), mutant plants displayed a delayed senescence phenotype, whereas plants overexpressing *OsNAC002* showed an early senescence [119]. In fact, OsNAC002 can bind the promoter of *CCGs* and upregulate their expression [119]. Further analyses may partially disclose OsNAC002 regulatory pathways. Ma et al. reported elevated *OsNAC002* expression in OsEIN2 overexpressing lines and, by contrast, downregulated expression in *ein2* mutants [120].

Moreover, in another study, Mao et al. [119] reported that OsNAC002 overexpressing plants presented elevated transcription of ABA biosynthesis genes (*OsNCED3* and *OsZEP1*) and that OsNAC002 bound to the promoter of ABA biosynthesis genes. Interestingly, *OsNAC002* is upregulated by low levels of ABA and downregulated by high levels of these phytohormone, which indicates a feedback repression of *OsNAC002* [119].

On the other hand, Lee et al. evaluated the crosstalk between the JA and ethylene pathways in rice with *oscoi1b-1* (a homologous gene for COI1, a F-Box protein involved in the degradation of the JA receptor) knock-out mutants [121]. These plants showed a stay-green phenotype, with substantial retention of chlorophyll and photosynthetic capacity. In addition, this mutation resulted in lower expression of *OsNAC002* and *OsEIN3*. Altogether, these results provide insight into the ABA–NAC–CCG pathways and the crosstalk between JA and ethylene pathways in rice [120,121]. Moreover, *osnac002* plants have shown a functional stay-green phenotype, with an increase of 10% in the grain yield [119].

In this sense, *OsNAC002* and *OsNAP* are two strong candidates for rice breeding biotechnological tools. Furthermore, the other six NAC genes (*OsNAC005*, *OsNAC006*, *OsNAC009*, *OsNAC010*, *OsNAC011* and *ONAC106*) displayed an abiotic stress regulation function in rice with an impact on senescence [122,123,124,125]. ONAC011 is a clear promoter of senescence. Plants overexpressing *ONAC011* showed a precocious senescence phenotype, whereas knockdown plants showed decreased heading time and leaf senescence with high accumulation of chlorophyll. However, both scenarios (overexpressing and knockdown plants) resulted in a reduction in grain yield. *onac106* mutant plants showed a delated senescence phenotype and differential expression of key SAGs (*SGR*, *NYC1*, *OsNAC5*, *OsNAP*, *OsEIN3* and *OsS3H*). Moreover, yeast one-hybrid assays showed that ONAC106 binds to the promoter regions of *SGR*, *NYC1*, *OsNAC5* and *LPA1*. This suggests that ONAC106 negatively regulates leaf senescence. Hence, more studies are necessary to clarify the molecular mechanisms, as well as the role of these genes in leaf senescence in rice.

In addition to the grain yield, another feature of crucial significance in rice breeding programs is the nutritional quality of grains. For instance, Zn and Fe are essential micronutrients. Whereas Fe participates as a catalytic cofactor in multiple metabolic pathways, Zn is a key structural component of enzymes and TFs. However, rice cultivars have poor quantities of these minerals [126]. The mineral content in the grain is closely related to nitrogen recycling uptake during senescence [127]. OsNAC005, which increases during rice senescence, may participate in the regulation of mineral remobilization from leaves to the sink through the activation of metal-homeostasis genes [122]. Further experiments are essential to improve grain nutritional qualities in this crop.

### 4.2. Wheat

Wheat is the second most widely grown crop in the world (220 million ha) [128]. Wheat grain consumption accounts for 19% of the calories consumed worldwide [129]. This grain is rich in carbohydrates, minerals (e.g., Zn, Fe) and vitamins and has a higher protein content than other major cereals; all these features make it an important nutritional source [130,131,132]. Bread wheat (*Triticum aestivum*) has a hexaploid genome that combines three grass genomes: *Aegilops speltoides*, *Triticum urartu* and *Triticum tauschii* [132]. Consequently, any genetic improvement of this crop becomes extremely complex. Moreover, the discovery of the SAG function in wheat has not enough landmarks because of the differences between the NAC ortholog in wheat and *Arabidopsis* [111]. However, the nutritional requirements of N and minerals (Zn, Fe) in wheat promote the study of TF genes that can improve grain quality.

Despite its genome complexity, 150 different *Triticeae* species provide an important genetic resource for crop breeding [128]. Saidi et al. [133] identified 168 NAC genes in durum wheat (AABB genome) including *TtNAM-B1* (GPC-B1) and *TtNAM-B2* (GPC-B2). TtNAM-B1 is a nonfunctional NAC TF in durum and bread wheat. However, *TaNAM-B1*, a wild allele for *TtNAM*-B1, is associated with the distribution of nutrients between leaves and developing grains [134]. Lines carrying the *TaNAM-B1* gene exhibit an increase of 10% in the grain protein content (GPC) as well as in zinc and iron [134]. Interestingly, these plants also present early senescence [97].

On the other hand, its homologous genes in the hexaploid wheat *GPC-A1* and *GPC-D1* had a redundant role in the regulation of monocarpic senescence and nutrient remobilization [135,136]. NAC TFs mutations may delay senescence and inhibit nutrient recycling in the last stages of this process, although grain weight was unaffected [135,136]. These findings highlight the importance of knowing not only the function of the candidate gene, but also the contribution of its homoeologous, mainly in polyploid crops, where redundancy might be expected.

Another recently reported NAC gene, *TaNAC-S*, is a negative regulator of leaf senescence. Wheat plants overexpressing *TaNAC-S* displayed a functional stay-green phenotype with increased grain yields and grain protein concentration [101]. On the other hand, Borril et al. [111] described three groups of upregulated genes during late senescence stages in *Triticum aestivum*. The first group includes two genes with high homology to the *Arabidopsis NAP* gene (*TraesCS5A02G143200* and *TraesCS5B02G142100*). The second group contains two genes with putative identity to *ANAC082* (*TraesCS1A02G2466300* and *TraesCS1B02G77300*). Finally, one gene was an *ANAC090* ortholog (*TraesCS5A02G127200*), although, in this case, differences in the expression time and phylogenetic distance with *Arabidopsis* must be taken into account before inferred any functionality [111]. Hence, functional studies are essential to establish the role of wheat NAC genes in senescence.

### 4.3. Barley

Barley (*Hordeum vulgare* L.) is a worldwide important crop for animal fodder and for fermentation in beer or whisky industries. Although barley is considered the most tolerant crop to salt and drought stresses, it is prone to premature leaf senescence. This can reduce remobilization and recycling of mineral nutrients and nitrogen-containing molecules from the leaves to the rest of the plant and therefore can affect grain filling [38].

Christiansen and Gregersen [137] reported a relevant study describing senescence on barley flag leaves. Although no function for any TFs was tested, a microarray analysis revealed valuable information at the regulatory level. They reported the upregulation of several genes encoding NAC, bZIP, MYB, bHLH, AP2/EREBP and CCAAT transcription factor families throughout senescence. As described for other species, NAC family members were highly associated with senescence. The best candidate gene was *HvNAC026*, because of its upregulated expression in senescing leaves and its almost undetectable expression in non-senescing leaves. However, *HvNAC023*, *HvNAC027*, *HvNAC029* and *HvNAC030* may also be candidates because of their relatively early upregulation that tends to level off towards the last stages of senescence [137,138]. In addition, *HvNAC005* was reported as a positive senescence regulator, since its overexpression produced early senescence, reduced root mass and poor seed setting in barley transgenic lines [138,139]. Similarly, another transcriptional analysis showed an association of some TFs, like *HvNAC001*, *HvNAC013*, *HvWRKY12* and MYB, with leaf senescence and nitrogen remobilization in barley, although more studies are required [140]. Finally, a non-conventional TF named WHIRLY1 was reported as a main regulator of some stress-induced senescence processes. This gene, previously reported as a nuclear transcription factor involved in activation of SAs related genes, regulates senescence induced by high light irradiance and drought [141]. Barley *whirly1* plants delay leaf senescence by altering the expression of the well-characterized SAG *HvS40* [142,143]. However, more studies are necessary to postulate *WHIRLY1* as a candidate gene for barley breeding.

### 4.4. Maize

Maize (*Zea mays*) is also one of the most important agronomic crops worldwide, with more than one million tons per year [144]. This crop is very demanding at the post-anthesis stages when nutrients are remobilized mainly to maximize the number of reproductive structures and to improve seed development [145]. Nitrogen is particularly essential for corn grain development. Its uptake in roots as well as its relocation from leaves impact directly on grain quality [146,147]. The stay-green phenotype has been studied in corn for several decades, although the molecular mechanism remains unclear [148,149]. Some stay-green hybrids delay leaf senescence, which results in crop yield earnings, especially under drought conditions [98,150]. Regarding nitrogen availability, Ma and Dwyer [148,149] demonstrated that stay-green varieties had a higher nitrogen-use efficiency than the conventional hybrids [145,149,151]. In this sense, stay-green hybrids in maize not only improved yields by prolonging leave lifespan, but also by improving nitrogen-use efficiency [152].

Transcriptional studies performing RNAseq profiles under abiotic factors, like drought, have been evaluated and associated with leaf senescence and the stay-green trait in maize [153,154]. Many TF families, including MYB, bHLH, C2H2, NAC, AP2/EREBP and bZIP families, changed expression throughout senescence [155]. Zhang et al. [155] performed a transcriptional analysis of mature as well as early and late senescence leaves. In this study, 12 NAC genes showed differential expression from early senescence towards late senescence. Among these genes, the expression of *GRMZM2G104400* and *GRMZM2G475014* increased at early senescence stages, as reported for their orthologs, and well-known *SAGs*, *VNI2* and *ORE1* in *Arabidopsis*. Moreover, the expression of *GRMZM2G146380*, *GRMZM2G114850*, *GRMZM2G163251*, *GRMZM2G109627* and *GRMZM2G011598* greatly increased at early and late stages of senescence, although no orthologs were reported in *Arabidopsis*. Nevertheless, these TFs represent interesting candidates for further characterizations in maize [155].

Recently Zhang et al. [156] reported a novel QTL controlling functional stay-green traits in maize through the evaluation of senescence-contrasting hybrid lines. In this study, the QTL was mapped to a single candidate gene called *NAC007*, which resulted to be one of the genes previously reported by Zhang et al. [155], *GRMZM2G114850*. To evaluate its function, Zhang et al. diminished *NAC007* expression by RNAi in two independent maize lines. The *NAC007* silenced lines showed delayed senescence and increased both nitrogen accumulation and biomass in vegetative tissues. These findings confirmed that these NAC negatively regulate the stay-green trait in maize. Moreover, the silenced maize lines were crossed with elite inbred testers resulting in stay-green hybrids with delayed leaf senescence. Indeed, these lines were grown in different field trials over two years in multiple locations and showed increased seed yield by an average of 0.29 megagram/hectare (4.6 bushel/acre). Furthermore, physiological and molecular measurements suggest that NAC007 has an essential role in regulating nutrients allocation from vegetative source to reproductive sink tissues [156].

Although Zhang et al. and Zhang et al. [155,156] described a practical example were the modulation of these genes may contribute to the stay-green trait, these types of studies in maize are still scarce. The available gene expression data regarding maize senescence could provide a useful source of information for generating new stay-green phenotypes in maize [157].

### 4.5. Sorghum

Sorghum (*Sorghum bicolor*) is a major source of food, especially for the underdeveloped regions or low-income countries, and may be an ideal biofuel crop for marginal lands [158]. Some sorghum lines show delayed leaf senescence. However, the knowledge of molecular mechanisms controlling this process is still limited [159]. To date, Wu et al. [159] presented a transcriptome profiling using RNAseq of developmental leaf senescence in which many of the detected TFs were orthologs of SAGs from other species. However, they did not report any proved functionality of these genes. Among the reported TFs, five families, NAC, ERF, WRKY, HSF and CO-like, were significantly overrepresented during sorghum leaf senescence. Regarding the NAC family, 16 genes corresponded to proteins whose sequences had similarity with AtORE1 and six of them increased their expression along early, mid and/or late senescence. Among these, Sb01g036590 had the highest expression and the highest protein similarity to ORE1. Thus, it is a potential candidate gene.

Another study has reported transcription analysis of dark- and drought-induced senescence [159]. Even though the study does not provide much information about NAC genes, it presents a possible candidate gene, *Sb01g006410*. This NAC is a middle-senescence marker gene whose expression increases throughout natural senescence and senescence induced by dark and drought. *Sb01g006410* is an ortholog to the negative regulator of senescence *Arabidopsis JUB1* [45,159].

### 4.6. Soybean

Soybean (*Glycine max*) is an oil crop in which the oil–protein balance in the grain is an important criterion of quality. An oil–protein proportion of 22%/42% in grains is good for producing flour (60% of the grain value) [160,161]. The high protein and essential amino acid contents make soybean one of the most important crops, with a production of 317 MMt per year [162]. Nevertheless, soybean is relatively poor in some amino acids like methionine (Met), cysteine (Cys), threonine (Thr) and lysine (Lys) [163]. Hence, the need to increase protein and sulfur amino acid content in seeds is one of the major goals in soybean breeding.

Of the 180 genes of the NAC TF families in soybean, almost half (44%) change expression during leaf senescence. Furthermore, most of the differentially expressed genes (90%) belong to the ATAF-like family [164]. Melo et al. [164] reviewed some differences and similarities of NAC family gene expression between soybean and *Arabidopsis*.

NAC TFs associated with senescence or cell death in soybean were GmNAC1, GmNAC5 and GmNAC6 (recently designated GmNAC81) [165,166,167]. These three genes were upregulated during senescence and the corresponding proteins localized in the nucleus. Moreover, its transient expression in tobacco leaves induced necrosis and enhanced the expression of senescence markers. *GmNAC1* has 67% identity with *NAP* and is upregulated by ABA treatment [166]. Transient expression of *GmNAC065* and *GmNAC085* genes in tobacco leaf induces chlorophyll loss, leaf yellowing, lipid peroxidation and H_2_O_2_ accumulation [164]. Besides, a yeast transactivation assay suggested co-expression of *GmNAC065* and *GmNAC085* form heterodimers, although they have opposite expression patterns during leaf senescence. *GmNAC65* is upregulated in the onset of senescence, whereas *GmNAC85* is downregulated at this point [164]. Furthermore, *GmNAC065* is a putative *VNI2* orthologue, whereas *GmNAC085* is the most closely related to *ANAC072*. However, they are expected to display distinct functional roles especially during leaf senescence [164].

GmNAC81 is a member of the subgroup TERN (tobacco elicitor-responsive gene–encoding NAC domain protein), which has a 62% identity with ANAC036 [168,169]. Soybean plants overexpressing *GmNAC81* showed an early senescence phenotype, whereas a reduced *GmNAC81* expression, through virus-induced gene silencing, delayed leaf senescence [170]. Furthermore, the overexpressing plants showed elevated levels of ABA and lower levels of SA during leaf senescence onset [170]. The results suggest that GmNAC81 may regulate senescence by altering ABA and SA biosynthesis under normal growth conditions [170]. Moreover, GmNAC81 seems to be involved in transducing cell death signal generated by ER (reticulum endoplasmic) and osmotic stress, which are induced by NRP-mediated cell death signaling pathway [167,168,171]. Other studies have shown that GmNAC81 acts downstream of NRP and that it can bind to common cis-regulatory sequences in target promoters like VPE (CASPASE-1–LIKE VACUOLAR PROCESSING ENZYME) to activate VPE gene expression [171]. Indeed, VPE participates in cell death, when triggered by pathogen infection, and is highly expressed in leaf senescence [172]. This pathway was originally identified in soybean and recently reported in *Arabidopsis*. Moreover, other NAC genes may collaborate with GmNAC81 in the activation of VPE. For example, GmNAC30 can form heterodimers with GmNAC81 and therefore bind to the promoter of VPE [171]. GmNAC30 shows highly conserved similarity with ATAF1 and ATAF2 and this makes it a strong candidate gene of the senescence regulation network in soybean [164].

### 4.7. Sunflower

Sunflower (*Helianhtus annuus* L.) is the fourth most important oil crop worldwide and produces high-quality oil for human consumption, and is also an important source of biodiesel [173]. Precocious senescence leads to economic losses in sunflower [174,175]. According to a detailed carbon (C) source–sink analysis during the pre-anthesis period, the contribution of fixed C was around 15% and 27% of the total carbon (i.e., fixed C + respired C) uptake of the grain in irrigated and in water-stressed crops, respectively [176]. In this sense, senescence in sunflower was linked to mainly water stress [23,177]. Besides, senescence is accelerated by nitrogen deficiency [178] and increased by light exposure during growth [179]. Plants grown under a high concentration of N (nitrate 20 mM) showed less decline in photosynthesis activity and a significant increase in the hexose to sucrose ratio at the onset of senescence than the plants grown under a low concentration of N [178]. The concentration of CO_2_, on the other hand, also promotes leaf senescence in sunflower, probably by affecting the soluble sugar levels, the C/N ratio and the oxidative status during leaf ontology [179].

Several studies have reported TFs regulating leaf senescence in sunflower. Among these TFs, the HD-Zip HaHB-4 has been widely used for crop breeding [180]. Ethylene positively regulates HaHB-4 during normal leaf senescence; once induced, HaHB-4 negatively regulates the biosynthesis of ethylene and the expression of genes related to this signaling pathway. Later, an expression gene analysis reported *HaNAC001* as the putative sunflower orthologous gene of *ORE1* [21]. HaNAC TFs are the third family of TFs most highly expressed during senescence in sunflower [99]. *HaNAC001* and *HaEIN2* transcription profiles during natural senescence showed an earlier upregulation in leaves of plants close to maturity, in comparison with young leaves of plants at the pre-anthesis stages [21].

On the other hand, *HaNAC002*, *HaNAC003* and *HaNAC005*, which are highly similar to *Arabidopsis ANAC072*, *ANAC055* and *ANAC019*, respectively, showed contrasting expression profiles during natural senescence. In *Arabidopsis*, *ANAC072*, *ANAC055* and *ANAC019* belong to the same clade of NAC genes and have overlapping expression patterns [181]. In sunflower, the expression of *HaNAC003* and *HaNAC005* rapidly increased towards anthesis. However, *HaNAC002* showed an opposite expression pattern.

*HaNAC004*, which is highly similar to *ANAC047*, was upregulated during leaf development in sunflower [21]. In *Arabidopsis*, *ANAC047* was upregulated during leaf senescence and downregulated in mutants with defective JAs, SAs or ethylene pathways, which suggests that this protein participates during leaf senescence in association with hormone signaling [24,182].

Recently, a gene network analysis (WGCNA) and a BioSignature Discoverer analysis of transcriptomic and metabolomics data provided a useful tool for identifying candidate genes and metabolites with a role during the triggering and development of leaf senescence in sunflower [22]. The results of these analyses showed that *HeAn_C_11118* (NCBI: NC_035440) is a key node in senescence. This NAC TF has high sequence similarity with *AtNAP*. The authors also reported a potential role of *HaNAC003* as a hub gene in leaf senescence. Furthermore, *HaNAC001*, *HaNAC003* and *HaNAC005* were also upregulated during senescence in an early senescence genotype in relation to a stay-green sunflower genotype [183].

### 4.8. Cotton

Cotton (*Gossypium hirsutum* L.) is one of the most important fiber production crops worldwide. The reduction in yield and fiber quality of this crop due to premature leaf senescence occurs with an increased frequency in many producing countries and causes important economic losses (up to 10% or 20% of total production in some cases) [184,185]. Lin et al. [186] reported a transcriptome analysis of cotton leaf senescence and identified 519 TFs from different families among all the differentially expressed genes. NAC, WRKY, bHLH, MYB, C3H, GRAS, DBP and AP2-EREBP were the most overrepresented TF families, with most of their members acting at early and mid-stages of the senescence process. *GhNAC12*, ortholog to the positive senescence regulator *ANAC059/ORS1* gene, is upregulated during leaf senescence and this leads to early senescence [187]. Moreover, a NAP-like transcription factor was associated with leaf senescence regulation. Ectopic expression of *GhNAP* can rescue the null *atnap* phenotype in *Arabidopsis*, and the GhNAPi lines of cotton present delayed leaf senesce, with no alteration of other agronomic traits. In comparison with the wild-type cotton, transgenic lines with reduced levels of *GhNAP* increased by 15% the lint yield, presumably by regulating secondary cell wall biosynthesis and deposition [188]. As reported for its ortholog in other species, a delay in leaf senescence was associated with regulation of different ABA pathways, which suggests that the function of NAP-like genes may be conserved over plant species [189].

Besides, different TF families regulate leaf senescence in cotton. Elasad et al. [190] reported nine cotton genes that increased their expression during leaf senescence, with *CotAD_37422* as the only TF member of the GRF (ortholog to the AT4G37740.1 gene). Further analyses must be done to confirm the functionality of this TF in regulating leaf senescence [190].

Finally, *GhWRKY27* (accession KF669775), a gene of the group III WRKY family, was recently reported in cotton. *GhWRKY27* was upregulated during early stages of senescence and its expression differs across different cotton lines. Indeed, *GhWRKY27* expression was higher in an early-ageing cotton variety than in a late-ageing cotton variety. The ectopic overexpression of *GhWRKY27* promoted leaf senescence in *Arabidopsis*. In addition, a yeast one-hybrid assay and electrophoretic mobility shift assay showed that GhWRKY27 directly binds to the promoter of a member of the cytochrome P450 family (GhCYP94C1), whose members are involved in leaf senescence [191,192].

### 4.9. Grapevine

Grapevine (*Vitis vinifera*) is one of the most important fruit crops because of its economic and agronomical inherence [110]. Leaf senescence seriously affects photosynthesis and nutrient assimilation, and thereby influences in yield and quality of grapes [193]. Some research has been made since the release of its genome in 2007, although the identification of TFs governing leaf senescence was poorly reported [194].

One of the few reports was the characterization of *VvNAC1*, a member of the NAP superfamily. In this report, the researchers analyzed the role of *VvNAC1* in development and in stress responses in grapevine and demonstrated that *VvNAC1* has a strong expression pattern at late stages in three organs (leaf, flower and berries). This finding indicates VvNAC1 may be a strong candidate in regulating developmental senescence, although no functionality was proved [195].

Recently, a member of the NAC family, DRL1, was reported as a main regulator in ABA-associated leaf senescence. The expression level of *DRL1* was higher in young leaves and decreased consistently with senescence progression. In other tissues, including stem flower and berry, the transcripts of *DRL1* remained at a very low level. Ectopic overexpression of *DRL1* in tobacco lines showed a delay in leaf senescence, suggesting that DRL1 negatively regulates leaf senescence presumably by altering ABA-signaling pathways [196]. Hence, this study showed how NAC family members not always act as positive actors of senescence progression, thus revealing new insights in negative regulation paths, as previously described for the *Arabidopsis* NAC transcription factor JUNGBRUNNEN1 and VNI2 [45,46].

### 4.10. Ornamental Species

Many efforts were achieved to genetically improve ornamental traits in several cultivars, with a main focused interest on carnation, rose, chrysanthemum and petunia. Such genetic modifications lead to distinct developmental and structural changes, like flower color, compact branching of internodes, flower longevity improvement, adventitious root development, changing flowering time and resistance to different biotic or abiotic stresses [197,198,199,200]. However, delaying of leaf senescence, which is an essential criterion of plant quality according to many ornamental breeders, has not been considered along these species.

Recently, Trupkin et al. [201] characterized the expression pattern of some NAC transcription factors during leaf and petal senescence progression in petunia *(Petunia hybrid*) and provided important information for future petunia genetic breeding programs. As expected, orthologs of the *AtORE1* and *AtNAP* genes in petunia were selected as the best candidate genes governing leaf and flower senescence, even though no functionality was proved [201].

## 5. Expression Pattern and Function Integration of NACs TFs Involved in Leaf Senescence across Species

NAC function, as well as different developmental processes in which they are involved, may have been conserved throughout evolution in several plant species, particularly between monocotyledonous and dicotyledonous plants [138,202,203,204,205]. However, divergent evolutionary patterns of NAC TFs, like the evolutionary rate of gene duplication and loss, have been reported between dicots and monocots by Jin et al. [206]. Therefore, to have a better understanding of NAC TFs acting as modulators of leaf senescence across species, we resumed and listed all TFs cited in this review to compare their expression pattern and functionality (Table 1).

For the NAC family, expression pattern analysis as an initial characterization method is a good starting point for selection of candidate genes as putative regulators of senescence processes. Despite some differences, NAC functionality was highly conserved throughout evolution. For example, putative orthologs of the *Arabidopsis NAP* gene seems to be central senescence regulators. Orthologues to NAP were found associated to leaf senescence in rice, sunflower, wheat, barley, soybean, grape, cotton and petunia (Table 1). NAP-like genes in those species showed a SAG behavior and increased their expression throughout leaf ontogeny. Furthermore, as described earlier, OsNAP and GhNAP has very similar functionality to AtNAP in the senescence process and are considered excellent candidates for the breeding process associated with a functional stay-green phenotype and enhanced agronomic traits [116,188,189]. Moreover, in barley and soybean, AtNAP orthologous promote leaf senescence although no functional stay-green lines were developed to assess crop productivity [139,166]. Hence, NAP-like TFs are putative targets for future breeding programs in outstanding agronomic relevant crops in almost all cited species, both monocots and dicots.

Another example is ORE1, one of the NAC TFs more widely characterized in *Arabidopsis*. Its regulation is based in ethylene activity during natural senescence in an age-dependent pathway [207]. Rice *osnac002* lines show a functional stay-green phenotype, with an increase of 10% in the grain yield. Moreover, ORE1 orthologues increased their expression along leaf ontogeny in rice, maize, sorghum, sunflower and petunia (Table 1). In this sense, ORE1 is a key TF in natural senescence and is considered a powerful candidate gene for crop breeding across species. Similarly, JUB1 and ATAF orthologues showed a conserved expression pattern across all cited species, regardless of monocots or dicots (Table 1). All this evidence suggests that some NAC TFs may have a conserved function in regulating leaf senescence throughout evolution.

By contrast, some other NAC members may differ in their expression pattern or functionally across species. *VNI2* expression increased in *Arabidopsis*, soybean and maize, but presented a different function on senescence regulation in *Arabidopsis* and soybean, both dicots. On the other hand, *ANAC100* is a homolog to ORE1 and is up-regulated in *Arabidopsis*, sunflower and barley, but delay senescence in rice (Table 1). Interestingly, *ANA072*, which is upregulated in *Arabidopsis* and rice, is downregulated in soybean and sunflower and, despite the difference in the expression pattern, this gene promotes leaf senescence in both *Arabidopsis* and soybean, again two dicot species (Table 1). As described before, the study of ANA072 is complex since it has high sequence identity to ANAC019 and ANAC055. Thus, the divergent result cited in this review could be explained by this redundancy [73].

Furthermore, the ANAC025 ortholog (TaNAMs) in wheat and ANAC022 orthologs (ONAC011 and ZmNAC007) in rice and maize positively regulates leaf senescence (Table 1). These two genes were not reported to be closely involved in leaf senescence in *Arabidopsis* or in any dicot species cited in this review. This finding suggests they could be regulators of senescence, specifically in monocot species. Furthermore, *ANAC036* increases its expression and promotes leaf senescence in soybean but decreases its expression and delays senescence in grapevine (Table 1). This gene was only reported in dicots species, although it was not described to regulate leaf senescence in *Arabidopsis*.

The divergent functionality of some NAC members across species may be explained by the number of genes gained in the dicot lineages throughout evolution, which was much larger than in the grass lineages [207]. Besides, phylogenetic distance in conjunction with ploidy number and differences in the life cycle (monocarpic or perennial cycle) between model species and crops can result in important differences at the molecular regulation. Such regulation may explain to some extent the difference in functionality between some NAC putative orthologues. Nevertheless, these previous studies in model species like *Arabidopsis* (dicot) and rice (monocot) were essential for the identification of molecular components regarding senescence process in non-model species, where genome complexity may hinder gene networks analysis [99]. Although, some examples like the detection of *TaNAM* in wheat from mutant varieties [97], *ZmNAC007* in stay-green varieties of maize [156] or the new stress-ER-induced senescence network in soybean [171] show the importance of continuing with conventional breeding in crop species.

## 6. Conclusions

Crop leaf senescence and grain yields have an inverse relation; that is, early senescence causes substantial biomass decrease. That is why working on plant senescence traits may result in crop yields improvements.

In this work, we discussed some practical examples regarding the importance of transcription factors acting as hub genes in senescence pathways. To our knowledge, stay-green genotypes were successfully selected in relevant agronomic crops like rice, wheat, maize and cotton, with direct improvements in yield or grain quality. Moreover, stay-green genotypes developed in some crops (barley, soybean, sunflower and sorghum) displayed improved agronomic traits, like stress tolerance or increased lifespan. Although, as described before, the improvement on yield or grain nutritional quality requires that the stay-green genotypes are accompanied by an efficient nutrient recycling and transport system from the source tissues to the grains once senescence is triggered.

This review compiles the overall knowledge of TFs, especially the NAC family, associated with leaf senescence available to date in the literature. New NGS technologies combined with molecular studies done in *Arabidopsis* serve as excellent kick-off information for generating stay-green genotypes in most agronomic crops. However, these previous studies cannot be transferred directly to all crops because senescence may have a different complex origin in other species or because this process may be intimately associated with a particular stress condition that has not been evaluated yet. As summarized in Table 1, putative orthologs from dicots and monocots have some similarities as well as some differences, even between classes. Therefore, as proposed by VanDerBuschel et al. [208], the appearance of new agronomic model plants that may share more molecular path signals with agronomic crops, is essential.

Indeed, the stay-green phenotype may be considered as one of the most promising traits in crop breeding programs, as it could diminish yield losses of plants growing in unfavorable environmental conditions. Functional stay-greens could provide an increase in the grain yield and an improvement in the nutrient content quality, although some studies should be carried over in order to assess if there is any correlation between agronomic traits like the number of reproductive structures, post-harvest senescence and functional stay-green lines in non-conventional crops. As a consequence, it should be considered that some SAG TFs might promote senescence and thus can improve grain yield and quality [97]. This strong impact may be due to their involvement in the recycling/transport mechanisms of nutrients that might impact in the grain filling process. Finally, we propose that cosmetic stay-green genotypes could be useful in breeding programs for most ornamental or horticultural plant species, where maintenance of greenery is an important quality trait.

## Figures and Tables

**Table 1:** Summary of the TFs involved in leaf senescence from different species. Expression and functional annotations are listed. Putative orthologues were obtained via BLASTp from the NCBI database.

Species	Code ID/Name	Orthologue in *A. thaliana*	Expression in Leaf Senescence	Function	Reference
*Arabidopsis thaliana*	AT1G01720/*ATAF1*	-	Increase	Promote leaf senescence	[44]
	AT1G52890/*ANAC019*	-	Increase	Promote leaf senescence	[73]
	AT3G15500/*ANAC055*	-	Increase	Promote leaf senescence	[73]
	AT4G27410*/ANAC072*	-	Increase	Promote leaf senescence	[73]
	AT1G34180*/ANAC016*	-	Increase	Promote leaf senescence	[43]
	AT1G69490/*NAP*	-	Increase	Promote leaf senescence	[31]
	AT5G13180/*VNI2*	-	Increase	Delay leaf senescence	[46]
	AT5G39610/*ORE1*	-	Increase	Promote leaf senescence	[17]
	AT2G38440/*ORS1*	-	Increase	Promote leaf senescence	[30]
	AT2G43000/*JUB1*	-	Increase	Delay leaf senescence	[45]
*Oryza sativa*	Os04g0460600/*OsNAC002*	*ORE1*	Increase	Promote leaf senescence	[119]
	Os01g0884300/*OsNAC006*	*ANAC072*	Increase	unclear	[123]
	Os03g21060/*OsNAP*	*NAP*	Increase	Promote leaf senescence	[113]
	Os11g0184900/*OsNAC005*	*ATAF1*	Increase	unclear	[113]
	Os03g0815100/*OsNAC009*	*NAP*	Increase	unclear	[122]
	Os11g03300/*OsNAC010*	*NAP*	Increase	unclear	[122]
	Os06g0675600*/ONAC011/OsY37*	*ANAC022*	Increase	Promote leaf senescence	[124]
	Os08g0433500/*ONAC106*	*ANAC100*	Decrease	Delay leaf senescence	[125]
*Triticum aestivum*	*TaNAM-A1 (GPC-A1)*	*ANAC025*	Increase	Promote leaf senescence	[136]
	*TaNAM-D1 (GPC-D1)*	*ANAC025*	Increase	Promote leaf senescence	[136]
	*TaNAM-B1 (GPC-B1)*	*ANAC025*	Increase	Promote leaf senescence	[97]
	*TaNAC-S*	*ANAC001*	Decrease	Delay leaf senescence	[101]
	TraesCS5A02G143200	*NAP*	Increase	unclear	[111]
	TraesCS5B02G142100	*NAP*	Increase	unclear	[111]
	TraesCS1A02G2466300	*ANAC082*	Increase	unclear	[111]
	TraesCS1B02G77300	*ANAC082*	Increase	unclear	[111]
	TraesCS5A02G127200	*ANAC090*	Increase	unclear	[111]
*Hordeum vulgare*	*HvNAC026*	*ANAC104*	Increase	unclear	[138]
	*HvNAC023*	*NAP*	Increase	unclear	[138]
	*HvNAC027*	*ANAC025*	Increase	unclear	[138]
	*HvNAC029*	*ANAC025*	Increase	unclear	[138]
	*HvNAC030*	*ANAC018*	Increase	unclear	[138]
	*HvNAC005*	*NAP*	Increase	Promote leaf senescence	[139]
	*HvNAC001*	*ANAC022*	Increase	unclear	[137]
	*HvNAC013*	*ANAC100*	Increase	unclear	[137]
	*HvWRKY12*	*AtWRKY018*	Increase	unclear	[140]
*Zea mays*	GRMZM2G104400	*VNI2*	Increase	unclear	[155]
	GRMZM2G475014	*ORE1*	Increase	unclear	[155]
	GRMZM2G146380	*ANAC046*	Increase	unclear	[155]
	GRMZM2G163251	*JUB1*	Increase	unclear	[155]
	GRMZM2G109627	*ANAC047*	Increase	unclear	[155]
	GRMZM2G011598	*ANAC025*	Increase	unclear	[155]
	GRMZM2G114850/*ZmNAC007*	*ANAC022*	Increase	Promote leaf senescence	[156]
*Sorghum bicolor*	Sb01g036590	*ORE1*	Increase	unclear	[159]
	Sb01g006410	*JUB1*	Increase	unclear	[159]
*Glycine max*	Gm0266x00007/*GmNAC1*	*NAP*	Increase	Promote leaf senescence	[166]
	Gm0025x00889/*GmNAC5*	*ANAC079*	Increase	Promote leaf senescence	[166]
	Glyma.08G360200/*GmNAC065*	*VNI2*	Increase	Promote leaf senescence	[164]
	Glyma.12G149100/*GmNAC085*	*ANAC072*	Decrease	Promote leaf senescence	[164]
	Glyma.12G022700/*GmNAC81*	*ANAC036*	Increase	Promote leaf senescence	[170]
	Glyma.05G195000/*GmNAC30*	*ATAF*	Increase	unclear	[164]
*Helianhtus annuus*	HanXRQChr13g0397761/*HaNAC001*	*ANAC100*	Increase	unclear	[21]
	HanXRQChr13g0407321/*HaNAC002*	*ANAC072*	Decrease	unclear	[21]
	HanXRQChr11g0327521/*HaNAC004*	*ANAC047*	Increase	unclear	[21]
	HanXRQChr03g0079641/*HaNAC005*	*ANAC019*	Increase	unclear	[21]
	HanXRQChr08g0210751/*HaNAC003*	*NAP*	Increase	unclear	[21]
	Han003584*/HaHB-4*	*AtHB7*	Increase	Delay leaf senescence	[180]
*Gossypium hirsutum*	*GhNAC12*	*ORS1*	Increase	Promote leaf senescence	[187]
	*GhNAP*	*NAP*	Increase	Promote leaf senescence	[188]
	CotAD_37422	*GRF2*	Increase	unclear	[190]
	KF669775/*GhWRKY27*	*AtWRKY041*	Increase	Promote leaf senescence	[191]
*Vitis vinifera*	*VvNAC1*	*NAP*	Increase	unclear	[195]
	XP-002281816/*VvDRL1*	*ANAC036*	Decrease	Delay leaf senescence	[196]
*Petunia hybrida*	Comp559557_c0_seq1/*PhNAC024*	*NAP*	Increase	unclear	[201]
	Comp22005_c0_seq3/*PhNAC017*	*ORE1*	Increase	unclear	[201]

## References

[1] Buchanan-Wollaston V., Earl S., Harrison E., Mathas E., Navabpour S., Page T., Pink D. (2003). The molecular analysis of leaf senescence—A genomics approach. Plant Biotechnol. J..

[2] Balazadeh S., Wu A., Mueller-Roeber B. (2010). Salt-triggered expression of the ANAC092-dependent senescence regulon in *Arabidopsis thaliana*. Plant Signal. Behav..

[3] Thomas H., Stoddart J.L. (1980). Leaf senescence. Annu. Rev. Plant Physiol..

[4] Woo H.R., Kim H.J., Lim P.O., Nam H.G. (2019). Leaf Senescence: Systems and Dynamics Aspects. Annu. Rev. Plant Biol..

[5] Thomas H., Huang L., Young M., Ougham H. (2009). Evolution of plant senescence. BMC Evol. Biol..

[6] Tegeder M., Rentsch D. (2010). Uptake and Partitioning of Amino Acids and Peptides. Mol. Plant.

[7] Tabbita F., Pearce S., Barneix A.J. (2017). Breeding for increased grain protein and micronutrient content in wheat: Ten years of the GPC-B1 gene. J. Cereal Sci..

[8] Yamaji N., Ma J.F. (2009). A Transporter at the Node Responsible for Intervascular Transfer of Silicon in Rice. Plant Cell.

[9] Patrick J.W., Offler C.E. (2001). Compartmentation of transport and transfer events in developing seeds. J. Exp. Bot..

[10] Pottier M., Daubresse C.M., Yoshimoto K., Thomine S. (2014). Autophagy as a possible mechanism for micronutrient remobilization from leaves to seeds. Front. Plant Sci..

[11] Maillard A., DiquÃlou S., Billard V., LaÃnÃ P., Garnica M., Prudent M., Garcia-Mina J.M., Yvin J.C., Ourry A. (2015). Leaf mineral nutrient remobilization during leaf senescence and modulation by nutrient deficiency. Front. Plant Sci..

[12] Park S.Y., Yu J.W., Park J.S., Li J., Yoo S.C., Lee N.Y., Lee S.K., Jeong S.W., Seo H.S., Koh H.J. (2007). The Senescence-Induced Staygreen Protein Regulates Chlorophyll Degradation. Plant Cell.

[13] Carrión C.A., Costa M.L., Martínez D.E., Mohr C., Humbeck K., Guiamet J.J. (2013). In vivo inhibition of cysteine proteases provides evidence for the involvement of ‘senescence-associated vacuoles’ in chloroplast protein degradation during dark-induced senescence of tobacco leaves. J. Exp. Bot..

[14] Martínez D.E., Costa M.L., Gomez F.M., Otegui M.S., Guiamet J.J. (2008). ‘Senescence-associated vacuoles’ are involved in the degradation of chloroplast proteins in tobacco leaves. Plant J..

[15] Breeze E., Harrison E., McHattie S., Hughes L., Hickman R., Hill C., Kiddle S., Kim Y.S., Penfold C.A., Jenkins D. (2011). High-Resolution Temporal Profiling of Transcripts during Arabidopsis Leaf Senescence Reveals a Distinct Chronology of Processes and Regulation. Plant Cell.

[16] Balazadeh S., Riaño-Pachón D.M., Mueller-Roeber B. (2008). Transcription factors regulating leaf senescence in *Arabidopsis thaliana*. Plant Biol..

[17] Balazadeh S., Siddiqui H., Allu A.D., Matallana-Ramirez L.P., Caldana C., Mehrnia M., Köhler B., Mueller-Roeber B., Zanor M.I. (2010). A gene regulatory network controlled by the NAC transcription factor ANAC092/AtNAC2/ORE1 during salt-promoted senescence. Plant J..

[18] Qiu K., Li Z., Yang Z., Chen J., Wu S., Zhu X., Gao S., Gao J., Ren G., Kuai B. (2015). EIN3 and ORE1 Accelerate Degreening during Ethylene-Mediated Leaf Senescence by Directly Activating Chlorophyll Catabolic Genes in Arabidopsis. PLoS Genet..

[19] Gil Nam H., Woo H.R., Kim J.H., Lim P.O. (2004). The Delayed Leaf Senescence Mutants of Arabidopsis, ore1, ore3, and ore9 are Tolerant to Oxidative Stress. Plant Cell Physiol..

[20] Matallana-Ramirez L.P., Rauf M., Farage-Barhom S., Dortay H., Xue G.P., Dröge-Laser W., Lers A., Balazadeh S., Mueller-Roeber B. (2013). NAC Transcription Factor ORE1 and Senescence-Induced BIFUNCTIONAL NUCLEASE1 (BFN1) Constitute a Regulatory Cascade in Arabidopsis. Mol. Plant.

[21] Moschen S., Luoni S.B., Paniego N.B., Hopp H.E., Dosio G.A.A., Fernandez P., Heinz R.A. (2014). Identification of candidate genes associated with leaf senescence in cultivated sunflower (*Helianthus annuus* L.). PLoS ONE.

[22] Moschen S., Higgins J., Di Rienzo J.A., Heinz R.A., Paniego N., Fernandez P. (2016). Network and biosignature analysis for the integration of transcriptomic and metabolomic data to characterize leaf senescence process in sunflower. BMC Bioinform..

[23] Moschen S., Di Rienzo J.A., Higgins J., Tohge T., Watanabe M., Rivarola M., Dopazo J., Hopp H.E., Hoefgen R., Fernie A.R. (2017). Integration of transcriptomic and metabolic data reveals hub transcription factors involved in drought stress response in sunflower (*Helianthus annuus* L.). Plant Mol. Biol..

[24] Kim H.J., Hong S.H., Kim Y.W., Lee I.H., Jun J.H., Phee B.K., Rupak T., Jeong H., Lee Y., Hong B.S. (2014). Gene regulatory cascade of senescence-associated NAC transcription factors activated by ETHYLENE-INSENSITIVE2-mediated leaf senescence signalling in Arabidopsis. J. Exp. Bot..

[25] Kim H.J., Nam H.G., Lim P.O. (2016). ScienceDirect Regulatory network of NAC transcription factors in leaf senescence. Curr. Opin. Plant Biol..

[26] Guo Y., Cai Z., Gan S. (2004). Transcriptome of Arabidopsis leaf senescence. Plant Cell Environ..

[27] Pruneda-Paz J.L., Breton G., Nagel D.H., Kang S.E., Bonaldi K., Doherty C.J., Ravelo S., Galli M., Ecker J.R., Kay S.A. (2014). A genome-scale resource for the functional characterization of Arabidopsis transcription factors. Cell Rep..

[28] Podzimska-Sroka D., O’Shea C., Gregersen P.L., Skriver K. (2015). NAC Transcription Factors in Senescence: From Molecular Structure to Function in Crops. Plants.

[29] Zentgraf U., Jobst J., Kolb D., Rentsch D. (2004). Senescence-Related Gene Expression Profiles of Rosette Leaves of *Arabidopsis thaliana*: Leaf Age Versus Plant Age. Plant Biol..

[30] Balazadeh S., Kwasniewski M., Caldana C., Mehrnia M., Zanor M.I., Xue G.P., Mueller-Roeber B. (2011). ORS1, an H_2_O_2_-Responsive NAC Transcription Factor, Controls Senescence in *Arabidopsis thaliana*. Mol. Plant.

[31] Guo Y., Gan S. (2006). AtNAP, a NAC family transcription factor, has an important role in leaf senescence. Plant J..

[32] Hu R., Qi G., Kong Y., Kong D., Gao Q., Zhou G. (2010). Comprehensive Analysis of NAC Domain Transcription Factor Gene Family in *Populus trichocarpa*. BMC Plant Biol..

[33] Jin H.K., Hye R.W., Kim J., Pyung O.L., In C.L., Seung H.C., Hwang D., Hong G.N. (2009). Trifurcate feed-forward regulation of age-dependent cell death involving miR164 in Arabidopsis. Science.

[34] Jaradat M.R., Feurtado J.A., Huang D., Lu Y., Cutler A.J. (2013). Multiple roles of the transcription factor AtMYBR1/AtMYB44 in ABA signaling, stress responses, and leaf senescence. BMC Plant Biol..

[35] Zhang L., Zhao G., Jia J., Liu X., Kong X. (2012). Molecular characterization of 60 isolated wheat MYB genes and analysis of their expression during abiotic stress. J. Exp. Bot..

[36] Phukan U.J., Jeena G.S., Tripathi V., Shukla R.K. (2017). Regulation of Apetala2/Ethylene Response Factors in Plants. Front. Plant Sci..

[37] Koyama T. (2014). The roles of ethylene and transcription factors in the regulation of onset of leaf senescence. Front. Plant Sci..

[38] Gregersen P.L., Culetic A., Boschian L., Krupinska K. (2013). Plant senescence and crop productivity. Plant Mol. Biol..

[39] Zhang H., Zhao M., Song Q., Zhao L., Wang G., Zhou C. (2016). Identification and function analyses of senescence-associated WRKYs in wheat. Biochem. Biophys. Res. Commun..

[40] Miao Y., Smykowski A., Zentgraf U. (2008). A novel upstream regulator ofWRKY53transcription during leaf senescence in *Arabidopsis thaliana*. Plant Biol..

[41] Ulker B., Mukhtar M.S., Somssich I.E. (2007). The WRKY70 transcription factor of Arabidopsis influences both the plant senescence and defense signaling pathways. Planta.

[42] Puranik S., Sahu P.P., Srivastava P.S., Prasad M. (2012). NAC proteins: Regulation and role in stress tolerance. Trends Plant Sci..

[43] Kim Y.S., Sakuraba Y., Han S.H., Yoo S.C., Paek N.C. (2013). Mutation of the Arabidopsis NAC016 Transcription Factor Delays Leaf Senescence. Plant Cell Physiol..

[44] Garapati P., Xue G.P., Munné-Bosch S., Balazadeh S. (2015). Transcription Factor ATAF1 in Arabidopsis Promotes Senescence by Direct Regulation of Key Chloroplast Maintenance and Senescence Transcriptional Cascades. Plant Physiol..

[45] Wu A., Allu A.D., Garapati P., Siddiqui H., Dortay H., Zanor M.I., Asensi-Fabado M.A., Munne-Bosch S., Antonio C., Tohge T. (2012). JUNGBRUNNEN1, a Reactive Oxygen Species-Responsive NAC Transcription Factor, Regulates Longevity in Arabidopsis. Plant Cell Online.

[46] Yang S.D., Seo P.J., Yoon H.K., Park C.M. (2011). The Arabidopsis NAC Transcription Factor VNI2 Integrates Abscisic Acid Signals into Leaf Senescence via the COR/RD Genes. Plant Cell.

[47] Iqbal N., Khan N.A., Ferrante A., Trivellini A., Francini A., Khan M.I.R. (2017). Ethylene Role in Plant Growth, Development and Senescence: Interaction with Other Phytohormones. Front. Plant Sci..

[48] Hunter D.A., Yoo S.D., Butcher S.M., McManus M.T. (2002). Expression of 1-Aminocyclopropane-1-Carboxylate Oxidase during Leaf Ontogeny in White Clover. Plant Physiol..

[49] Grbić V., Bleecker A.B. (1995). Ethylene regulates the timing of leaf senescence in Arabidopsis. Plant J..

[50] Jibran R., Hunter D.A., Dijkwel P.P. (2013). Hormonal regulation of leaf senescence through integration of developmental and stress signals. Plant Mol. Biol..

[51] Jing H.C., Schippers J.H.M., Hille J., Dijkwel P.P. (2005). Ethylene-induced leaf senescence depends on age-related changes and OLD genes in Arabidopsis. J. Exp. Bot..

[52] Kim J., Park S.J., Lee I.H., Chu H., Penfold C.A., Kim J.H., Buchanan-Wollaston V., Gil Nam H., Woo H.R., Lim P.O. (2018). Comparative transcriptome analysis in Arabidopsis ein2/ore3 and ahk3/ore12 mutants during dark-induced leaf senescence. J. Exp. Bot..

[53] Rauf M., Arif M., Dortay H., Matallana-Ramírez L.P., Waters M.T., Gil Nam H., Lim P.O., Mueller-Roeber B., Balazadeh S. (2013). ORE1 balances leaf senescence against maintenance by antagonizing G2-like-mediated transcription. EMBO Rep..

[54] Chang K.N., Zhong S., Weirauch M.T., Hon G., Pelizzola M., Li H., Huang S.S.C., Schmitz R.J., Urich M., Kuo D. (2013). Temporal transcriptional response to ethylene gas drives growth hormone cross-regulation in Arabidopsis. eLife.

[55] Alonso J.M., Hirayama T., Roman G., Nourizadeh S., Ecker J.R. (1999). EIN2, a Bifunctional Transducer of Ethylene and Stress Responses in Arabidopsis. Science.

[56] Lei G., Shen M., Li Z.-G., Zhang B., Duan K.-X., Wang N., Cao Y.-R., Zhang W.-K., Ma B., Ling H.-Q. (2011). EIN2 regulates salt stress response and interacts with a MA3 domain-containing protein ECIP1 in Arabidopsis. Plant Cell Environ..

[57] Lu P.L., Chen N.Z., An R., Su Z., Qi B.S., Ren F., Chen J., Wang X.C. (2007). A novel drought-inducible gene, *ATAF1*, encodes a NAC family protein that negatively regulates the expression of stress-responsive genes in Arabidopsis. Plant Mol. Biol..

[58] Wang Y., Liu C., Li K., Sun F., Hu H., Zhao Y., Han C., Zhang W., Duan Y., Liu M. (2007). Arabidopsis EIN2 modulates stress response through abscisic acid response pathway. Plant Mol. Biol..

[59] Hauser F., Waadt R., Schroeder J.I. (2011). Evolution of Abscisic Acid Synthesis and Signaling Mechanisms. Curr. Biol..

[60] Nakashima K., Yamaguchi-Shinozaki K. (2013). ABA signaling in stress-response and seed development. Plant Cell Rep..

[61] Asad M.A.U., Zakari S.A., Zhao Q., Zhou L., Ye Y., Cheng F. (2019). Abiotic Stresses Intervene with ABA Signaling to Induce Destructive Metabolic Pathways Leading to Death: Premature Leaf Senescence in Plants. Int. J. Mol. Sci..

[62] Sakuraba Y., Jeong J., Kang M.Y., Kim J., Paek N.C., Choi G. (2014). Phytochrome-interacting transcription factors PIF4 and PIF5 induce leaf senescence in Arabidopsis. Nat. Commun..

[63] Sakuraba Y., Kim Y.S., Han S.H., Lee B.D., Paek N.C. (2015). The Arabidopsis Transcription Factor NAC016 Promotes Drought Stress Responses by Repressing AREB1 Transcription through a Trifurcate Feed-Forward Regulatory Loop Involving NAP. Plant Cell.

[64] Yang J., Worley E., Udvardi M. (2014). A NAP-AAO_3_ Regulatory Module Promotes Chlorophyll Degradation via ABA Biosynthesis in Arabidopsis Leaves. Plant Cell.

[65] Seo M., Aoki H., Koiwai H., Kamiya Y., Nambara E., Koshiba T. (2004). Comparative Studies on the Arabidopsis Aldehyde Oxidase (AAO) Gene Family Revealed a Major Role of AAO_3_ in ABA Biosynthesis in Seeds. Plant Cell Physiol..

[66] Zhang K., Gan S.S. (2011). An Abscisic Acid-AtNAP Transcription Factor-SAG_113_ Protein Phosphatase _2_C Regulatory Chain for Controlling Dehydration in Senescing Arabidopsis Leaves. Plant Physiol..

[67] Guan C., Wang X., Feng J., Hong S., Liang Y., Ren B., Zuo J. (2014). Cytokinin Antagonizes Abscisic Acid-Mediated Inhibition of Cotyledon Greening by Promoting the Degradation of ABSCISIC ACID INSENSITIVE5 Protein in Arabidopsis. Plant Physiol..

[68] Schippers J.H.M. (2015). Science Direct Transcriptional networks in leaf senescence. Curr. Opin. Plant Biol..

[69] Sakuraba Y., Han S.H., Lee S.H., Hörtensteiner S., Paek N.C. (2016). Arabidopsis NAC_O1_6 promotes chlorophyll breakdown by directly upregulating *STAYGREEN_1_* transcription. Plant Cell Rep..

[70] Yoshida T., Fujita Y., Sayama H., Kidokoro S., Maruyama K., Mizoi J., Shinozaki K., Yamaguchi-Shinozaki K. (2010). AREB1, AREB2, and ABF3 are master transcription factors that cooperatively regulate ABRE-dependent ABA signaling involved in drought stress tolerance and require ABA for full activation. Plant J..

[71] Seok H.Y., Woo D.H., Nguyen L.V., Tran H.T., Tarte V.N., Mehdi SM M., Lee S.Y., Moon Y.H. (2017). Arabidopsis AtNAP functions as a negative regulator via repression of AREB1 in salt stress response. Planta.

[72] Jensen M.K., Lindemose S., De Masi F., Reimer J.J., Nielsen M., Perera V., Workman C.T., Turck F., Grant M.R., Mundy J. (2013). ATAF1 transcription factor directly regulates abscisic acid biosynthetic gene NCED3 in *Arabidopsis thaliana*. FEBS Open Bio.

[73] Hickman R., Hill C., Penfold C.A., Breeze E., Bowden L., Moore J.D., Zhang P., Jackson A., Cooke E., Bewicke-Copley F. (2013). A local regulatory network around three NAC transcription factors in stress responses and senescence in *Arabidopsis leaves*. Plant J..

[74] Lindemose S., Jensen M.K., Van De Velde J., O’Shea C., Heyndrickx K.S., Workman C.T., Vandepoele K., Skriver K., De Masi F. (2014). A DNA-binding-site landscape and regulatory network analysis for NAC transcription factors in *Arabidopsis thaliana*. Nucleic Acids Res..

[75] Bu Q., Jiang H., Li C.B., Zhai Q., Zhang J., Wu X., Sun J., Xie Q., Li C. (2008). Role of the *Arabidopsis thaliana* NAC transcription factors ANAC019 and ANAC055 in regulating jasmonic acid-signaled defense responses. Cell Res..

[76] Takasaki H., Maruyama K., Takahashi F., Fujita M., Yoshida T., Nakashima K., Myouga F., Toyooka K., Yamaguchi-Shinozaki K., Shinozaki K. (2015). SNAC-As, stress-responsive NAC transcription factors, mediate ABA-inducible leaf senescence. Plant J..

[77] Zhu X., Chen J., Xie Z., Gao J., Ren G., Zhou X., Kuai B. (2015). Jasmonic acid promotes degreening via MYC2/3/4- and ANAC019/055/072-mediated regulation of major chlorophyll catabolic genes. Plant J..

[78] Zheng X.Y., Spivey N.W., Zeng W., Liu P.P., Fu Z.Q., Klessig D.F., He S.Y., Dong X. (2012). Coronatine promotes Pseudomonas syringae virulence in plants by activating a signaling cascade that inhibits salicylic acid accumulation. Cell Host Microbe.

[79] Schweizer F., Bodenhausen N., Lassueur S., Masclaux F.G., Reymond P. (2013). Differential Contribution of Transcription Factors to *Arabidopsis thaliana* Defense Against *Spodoptera littoralis*. Front. Plant Sci..

[80] Fujita M., Fujita Y., Maruyama K., Seki M., Hiratsu K., Ohme-Takagi M., Tran L.S.P., Yamaguchi-Shinozaki K., Shinozaki K. (2004). A dehydration-induced NAC protein, RD26, is involved in a novel ABA-dependent stress-signaling pathway. Plant J..

[81] Tran L.S.P., Nakashima K., Sakuma Y., Simpson S.D., Fujita Y., Maruyama K., Fujita M., Seki M., Shinozaki K., Yamaguchi-Shinozaki K. (2004). Isolation and Functional Analysis of Arabidopsis Stress-Inducible NAC Transcription Factors That Bind to a Drought-Responsive *cis-Element* in the early responsive to dehydration stress 1 Promoter. Plant Cell.

[82] Li X., Li M., Yan Y., Liu X., Li L. (2016). Dual Function of NAC072 in Gene Regulation in Arabidopsis. Front. Plant Sci..

[83] Kim T.W., Guan S., Sun Y., Deng Z., Tang W., Shang J.X., Sun Y., Burlingame A.L., Wang Z.Y. (2009). Brassinosteroid signal transduction from cell surface receptor kinases to nuclear transcription factors. Nature.

[84] Chung Y., Kwon S.I., Choe S. (2014). Antagonistic Regulation of Arabidopsis Growth by Brassinosteroids and Abiotic Stresses. Mol. Cells.

[85] Ye H., Liu S., Tang B., Chen J., Xie Z., Nolan T.M., Jiang H., Guo H., Lin H.Y., Li L. (2017). RD26 mediates crosstalk between drought and brassinosteroid signalling pathways. Nat. Commun..

[86] Hu Y., Jiang Y., Han X., Wang H., Pan J., Yu D. (2017). Jasmonate regulates leaf senescence and tolerance to cold stress: Crosstalk with other phytohormones. J. Exp. Bot..

[87] Chini A., Gimenez-Ibanez S., Goossens A., Solano R. (2016). Redundancy and specificity in jasmonate signalling. Curr. Opin. Plant Biol..

[88] Wasternack C., Hause B. (2013). Jasmonates: Biosynthesis, perception, signal transduction and action in plant stress response, growth and development. An update to the 2007 review in Annals of Botany. Ann. Bot..

[89] Hickman R., Van Verk M.C., Van Dijken A.J.H., Mendes M.P., Vroegop-Vos I.A., Caarls L., Steenbergen M., Van der Nagel I., Wesselink G.J., Jironkin A. (2017). Architecture and Dynamics of the Jasmonic Acid Gene Regulatory Network. Plant Cell.

[90] Kim J., Chang C., Tucker M.L. (2015). To grow old: Regulatory role of ethylene and jasmonic acid in senescence. Front. Plant Sci..

[91] Li Z., Peng J., Wen X., Guo H. (2013). ETHYLENE-INSENSITIVE3 Is a Senescence-Associated Gene That Accelerates Age-Dependent Leaf Senescence by Directly Repressing miR164 Transcription in Arabidopsis. Plant Cell.

[92] Li S., Gao J., Yao L., Ren G., Zhu X., Gao S., Qiu K., Zhou X., Kuai B. (2016). The role of ANAC072 in the regulation of chlorophyll degradation during age-and dark-induced leaf senescence. Plant Cell Rep..

[93] Kamranfar I., Xue G.P., Tohge T., Sedaghatmehr M., Fernie A.R., Balazadeh S., Mueller-Roeber B. (2018). Transcription factor RD26 is a key regulator of metabolic reprogramming during dark-induced senescence. New Phytol..

[94] Chen Y., Wei J., Wang M., Shi Z., Gong W., Zhang M. (2012). The Crystal Structure of Arabidopsis VSP1 Reveals the Plant Class C-Like Phosphatase Structure of the DDDD Superfamily of Phosphohydrolases. PLoS ONE.

[95] Kim H.J., Park J.H., Kim J., Kim J.J., Hong S., Kim J., Kim J.H., Woo H.R., Hyeon C., Lim P.O. (2018). Time-evolving genetic networks reveal a NAC troika that negatively regulates leaf senescence in Arabidopsis. Proc. Natl. Acad. Sci. USA.

[96] Seo P.J., Park C.M. (2011). Signaling linkage between environmental stress resistance and leaf senescence in Arabidopsis. Plant Signal. Behav..

[97] Uauy C., Distelfeld A., Fahima T., Blechl A., Dubcovsky J. (2006). A NAC gene regulating senescence improves grain protein, Zn, and Fe content in wheat. Science.

[98] Thomas H., Smart C.M. (1993). Crops that stay green. Ann. Appl. Biol..

[99] Moschen S., Bengoa Luoni S., Di Rienzo J.A., Caro M.D., Tohge T., Watanabe M., Hollmann J., González S., Rivarola M., García-García F. (2016). Integrating transcriptomic and metabolomic analysis to understand natural leaf senescence in sunflower. Plant Biotechnol. J..

[100] Ding L., Wang K.J., Jiang G.M., Liu M.Z., Gao L.M. (2007). Photosynthetic rate and yield formation in different maize hybrids. Biol. Plant..

[101] Zhao D., Derkx A.P., Liu D.C., Buchner P., Hawkesford M.J. (2015). Overexpression of a NAC transcription factor delays leaf senescence and increases grain nitrogen concentration in wheat. Plant Biol..

[102] Cruickshank A.W., Borrell A.K., Henzell R., Jordan D.R., Hunt C.H. (2012). The Relationship between the Stay-Green Trait and Grain Yield in Elite Sorghum Hybrids Grown in a Range of Environments. Crop. Sci..

[103] Gustafson P., Lee S.H., Fu J.D., Yan Y.F., Kim M.Y., Lee B.W. (2011). Population-specific quantitative trait loci mapping for functional stay-green trait in rice (*Oryza sativa* L.). Genome.

[104] Hafsi M., Mechmeche W., Bouamama L., Djekoune A., Zaharieva M., Monneveux P. (2000). Flag Leaf Senescence, as Evaluated by Numerical Image Analysis, and its Relationship with Yield under Drought in Durum Wheat. J. Agron. Crop. Sci..

[105] Kusaba M., Tanaka A., Tanaka R. (2013). Stay-green plants: What do they tell us about the molecular mechanism of leaf senescence. Photosynth. Res..

[106] De La Vega A., Cantore M., Sposaro M., Trapani N., Pereira M.L., Hall A. (2011). Canopy stay-green and yield in non-stressed sunflower. Field Crop. Res..

[107] Luquez V.M., Guiamét J.J. (2002). The stay green mutations d1 and d2 increase water stress susceptibility in soybeans. J. Exp. Bot..

[108] Dohleman F.G., Long S.P. (2009). More Productive Than Maize in the Midwest: How Does Miscanthus Do It?. Plant Physiol..

[109] Zhao Y., Qiang C., Wang X., Chen Y., Deng J., Jiang C., Sun X., Chen H., Li J., Piao W. (2019). New alleles for chlorophyll content and stay-green traits revealed by a genome wide association study in rice (*Oryza sativa*). Sci. Rep..

[110] Wang N., Zheng Y., Xin H. (2013). Comprehensive analysis of NAC domain transcription factor gene family in *Vitis vinifera*. Plant Cell Rep..

[111] Borrill P., Harrington S.A., Simmonds J., Uauy C. (2018). Identification of transcription factors regulating senescence in wheat through gene regulatory network modelling. bioRxiv.

[112] Leng Y., Ye G., Zeng D. (2017). Genetic Dissection of Leaf Senescence in Rice. Int. J. Mol. Sci..

[113] Khush G.S. (2005). What it will take to Feed 5.0 Billion Rice consumers in 2030. Plant Mol. Biol..

[114] Anderson P.K., Cunningham A.A., Patel N.G., Morales F.J., Epstein P.R., Daszak P. (2004). Emerging infectious diseases of plants: Pathogen pollution, climate change and agrotechnology drivers. Trends Ecol. Evol..

[115] Chen X., Wang Y., Lv B., Li J., Luo L., Lu S., Zhang X., Ma H., Ming F. (2014). The NAC Family Transcription Factor OsNAP Confers Abiotic Stress Response Through the ABA Pathway. Plant Cell Physiol..

[116] Liang C., Wang Y., Zhu Y., Tang J., Hu B., Liu L., Ou S., Wu H., Sun X., Chu J. (2014). OsNAP connects abscisic acid and leaf senescence by fine-tuning abscisic acid biosynthesis and directly targeting senescence-associated genes in rice. Proc. Natl. Acad. Sci. USA.

[117] Zhou Y., Huang W., Liu L., Chen T., Zhou F., Lin Y. (2013). Identification and functional characterization of a rice NAC gene involved in the regulation of leaf senescence. BMC Plant Biol..

[118] Tang X., Zhang R., Chen X., Wu X., Ming F. (2014). Characterization of OsNAP from *Oryza sativa* L. and its application in molecular breeding. J. Fudan Univ. Nat. Sci..

[119] Mao C., Lu S., Lv B., Zhang B., Shen J., He J., Luo L., Xi D., Chen X., Ming F. (2017). A Rice NAC Transcription Factor Promotes Leaf Senescence via ABA Biosynthesis. Plant Physiol..

[120] Ma B., He S.J., Duan K.X., Yin C.C., Chen H., Yang C., Xiong Q., Song Q.X., Lu X., Chen H.W. (2013). Identification of Rice Ethylene-Response Mutants and Characterization of MHZ7/OsEIN2 in Distinct Ethylene Response and Yield Trait Regulation. Mol. Plant.

[121] Lee S.H., Sakuraba Y., Lee T., Kim K.W., An G., Lee H.Y., Paek N.C. (2015). Mutation of *Oryza sativa* CORONATINE INSENSITIVE 1b (OsCOI1b) delays leaf senescence. J. Integr. Plant Biol..

[122] Ricachenevsky F.K., Menguer P.K., Sperotto R.A. (2013). kNACking on heaven’s door: How important are NAC transcription factors for leaf senescence and Fe/Zn remobilization to seeds?. Front. Plant Sci..

[123] Chung P.J., Jung H., Choi Y.D., Kim J.-K. (2018). Genome-wide analyses of direct target genes of four rice NAC-domain transcription factors involved in drought tolerance. BMC Genom..

[124] El Mannai Y., Akabane K., Hiratsu K., Satoh-Nagasawa N., Wabiko H. (2017). The NAC Transcription Factor Gene OsY37 (ONAC011) Promotes Leaf Senescence and Accelerates Heading Time in Rice. Int. J. Mol. Sci..

[125] Sakuraba Y., Piao W., Lim J.H., Han S.H., Kim Y.S., An G., Paek N.C. (2015). Rice ONAC106 Inhibits Leaf Senescence and Increases Salt Tolerance and Tiller Angle. Plant Cell Physiol..

[126] Sperotto R.A., Ricachenevsky F.K., de Abreu Waldow V., Fett J.P. (2012). Iron biofortification in rice: It’s a long way to the top. Plant Sci..

[127] Kutman U.B., Yildiz B., Cakmak I. (2011). Effect of nitrogen on uptake, remobilization and partitioning of zinc and iron throughout the development of durum wheat. Plant Soil.

[128] Ortiz R., Braun H.-J., Crossa J., Crouch J.H., Davenport G., Dixon J., Dreisigacker S., Duveiller E., He Z., Huerta J. (2008). Wheat genetic resources enhancement by the International Maize and Wheat Improvement Center (CIMMYT). Genet. Resour. Crop Evol..

[129] Aksoy M.A., Beghin J.C. (2004). Global Agricultural Trade and Developing Countries.

[130] Zörb C., Langenkämper G., Betsche T., Niehaus K., Barsch A. (2006). Metabolite Profiling of Wheat Grains (*Triticum aestivum* L.) from Organic and Conventional Agriculture. J. Agric. Food Chem..

[131] Langenkämper G., Zörb C., Seifert M., Mäder P., Fretzdorff B., Betsche T. (2006). Nutritional quality of organic and conventional wheat. J. Appl. Bot. Food Qual..

[132] Shewry P.R. (2009). Wheat. J. Exp. Bot..

[133] Saidi M.N., Mergby D., Brini F. (2017). Identification and expression analysis of the NAC transcription factor family in durum wheat (*Triticum turgidum* L. ssp. durum). Plant Physiol. Biochem..

[134] Brevis J.C., Uauy C., Dubcovsky J. (2006). The high grain protein content gene Gpc-B1 accelerates senescence and has pleiotropic effects on protein content in wheat. J. Exp. Bot..

[135] Pearce S., Tabbita F., Cantu D., Buffalo V., Avni R., Vazquez-Gross H., Zhao R., Conley C.J., Distelfeld A., Dubcovksy J. (2014). Regulation of Zn and Fe transporters by the GPC1 gene during early wheat monocarpic senescence. BMC Plant Biol..

[136] Avni R., Zhao R., Pearce S., Jun Y., Uauy C., Tabbita F., Fahima T., Slade A., Dubcovsky J., Distelfeld A. (2014). Functional characterization of GPC-1 genes in hexaploid wheat. Planta.

[137] Christiansen M.W., Gregersen P.L. (2014). Members of the barley NAC transcription factor gene family show differential co-regulation with senescence-associated genes during senescence of flag leaves. J. Exp. Bot..

[138] Christiansen M.W., Holm P.B., Gregersen P.L. (2011). Characterization of barley (*Hordeum vulgare* L.) NAC transcription factors suggests conserved functions compared to both monocots and dicots. BMC Res. Notes.

[139] Christiansen M.W., Matthewman C., Podzimska-Sroka D., O’Shea C., Lindemose S., Møllegaard N.E., Holme I.B., Hebelstrup K., Skriver K., Gregersen P.L. (2016). Barley plants over-expressing the NAC transcription factor gene HvNAC005 show stunting and delay in development combined with early senescence. J. Exp. Bot..

[140] Hollmann J., Gregersen P.L., Krupinska K. (2014). Identification of predominant genes involved in regulation and execution of senescence-associated nitrogen remobilization in flag leaves of field grown barley. J. Exp. Bot..

[141] Foyer C.H., Karpinska B., Krupinska K. (2014). The functions of WHIRLY1 and REDOX-RESPONSIVE TRANSCRIPTION FACTOR 1 in cross tolerance responses in plants: A hypothesis. Philos. Trans. R. Soc. B Biol. Sci..

[142] Janack B., Sosoi P., Krupinska K., Humbeck K. (2016). Knockdown of WHIRLY1 Affects Drought Stress-Induced Leaf Senescence and Histone Modifications of the Senescence-Associated Gene HvS40. Plants.

[143] Kucharewicz W., Distelfeld A., Bilger W., Müller M., Munné-Bosch S., Hensel G., Krupinska K. (2017). Acceleration of leaf senescence is slowed down in transgenic barley plants deficient in the DNA/RNA-binding protein WHIRLY1. J. Exp. Bot..

[144] FAO FAOSTAT, Production. http://faostat3.fao.org/browse/Q/QC/E.

[145] Borrell A., Hammer G., Oosterom E. (2001). Stay-green: A consequence of the balance between supply and demand for nitrogen during grain filling?. Ann. Appl. Biol..

[146] Nasielski J., Earl H., Deen B., Deen B. (2019). Luxury Vegetative Nitrogen Uptake in Maize Buffers Grain Yield under Post-silking Water and Nitrogen Stress: A Mechanistic Understanding. Front. Plant Sci..

[147] Woli K.P., Sawyer J.E., Boyer M.J., Abendroth L.J., Elmore R.W. (2019). Corn Era Hybrid Nutrient Concentration and Accumulation of Secondary and Micronutrients. Agron. J..

[148] Tollenaar M., Ahmadzadeh A., Lee E.A. (2004). Physiological Basis of Heterosis for Grain Yield in Maize. Crop. Sci..

[149] Ma B., Dwyer L. (1998). Nitrogen uptake and use of two contrasting maize hybrids differing in leaf senescence. Plant Soil.

[150] Bekavac G., Purar B., Stojaković M., Jocković D., Ivanović M., Nastasić A. (2007). Genetic analysis of stay-green trait in broad-based maize populations. Cereal Res. Commun..

[151] Rajcan I., Tollenaar M. (1999). Source: Sink ratio and leaf senescence in maize: II. Nitrogen metabolism during grain filling. Field Crop. Res..

[152] Mu X., Chen Q., Chen F., Yuan L., Mi G. (2018). Dynamic remobilization of leaf nitrogen components in relation to photosynthetic rate during grain filling in maize. Plant Physiol. Biochem..

[153] Li P., Cao W., Fang H., Xu S., Yin S., Zhang Y., Lin D., Wang J., Chen Y., Xu C. (2017). Transcriptomic Profiling of the Maize (*Zea mays* L.) Leaf Response to Abiotic Stresses at the Seedling Stage. Front. Plant Sci..

[154] Shiriga K., Sharma R., Kumar K., Yadav S.K., Hossain F., Thirunavukkarasu N. (2014). Genome-wide identification and expression pattern of drought-responsive members of the NAC family in maize. Meta Gene.

[155] Zhang W.Y., Xu Y.C., Li W.L., Yang L., Yue X., Zhang X.S., Zhao X.Y. (2014). Transcriptional Analyses of Natural Leaf Senescence in Maize. PLoS ONE.

[156] Zhang J., Fengler K.A., Van Hemert J.L., Gupta R., Mongar N., Sun J., Allen W.B., Wang Y., Weers B., Mo H. (2019). Identification and characterization of a novel stay-green QTL that increases yield in maize. Plant Biotechnol. J..

[157] Campos H., Cooper M., Habben J., Edmeades G., Schussler J. (2004). Improving drought tolerance in maize: A view from industry. Field Crop. Res..

[158] Calviño M., Messing J. (2012). Sweet sorghum as a model system for bioenergy crops. Curr. Opin. Biotechnol..

[159] Wu X.Y., Hu W.J., Xia Y., Zhao Y., Wang L.D., Zhang L.M., Luo H., Luo J.C., Jing H.C. (2016). Transcriptome profiling of developmental leaf senescence in sorghum (*Sorghum bicolor*). Plant Mol. Biol..

[160] Pettersson D., Pontoppi K. (2013). Soybean Meal and The Potential for Upgrading Its Feeding Value by Enzyme Supplementation. Soybean Bio Act. Compd..

[161] Patil G., Mian R., Vuong T., Pantalone V., Song Q., Chen P., Shannon G.J., Carter T.C., Nguyen H.T. (2017). Molecular mapping and genomics of soybean seed protein: A review and perspective for the future. Theor. Appl. Genet..

[162] American Soybean Association SoyStats. http://www.soystats.com.

[163] Warrington C.V., Abdel-Haleem H., Hyten D.L., Cregan P.B., Orf J.H., Killam A.S., Bajjalieh N., Li Z., Boerma H.R. (2015). QTL for seed protein and amino acids in the Benning × Danbaekkong soybean population. Theor. Appl. Genet..

[164] Melo B.P., Fraga O.T., Silva J.C., Ferreira D.O., Brustolini O.J., Carpinetti P.A., Machado J.P., Reis P.A., Fontes E.P. (2018). Revisiting the Soybean GmNAC Superfamily. Front. Plant Sci..

[165] Meng Q., Zhang C., Gai J., Yu D. (2007). Molecular cloning, sequence characterization and tissue-specific expression of six NAC-like genes in soybean (*Glycine max* (L.) Merr.). J. Plant Physiol..

[166] Pinheiro G.L., Marques C.S., Costa M.D., Reis P.A., Alves M.S., Carvalho C.M., Fietto L.G., Fontes E.P. (2009). Complete inventory of soybean NAC transcription factors: Sequence conservation and expression analysis uncover their distinct roles in stress response. Gene.

[167] Faria J.A., Ab Reis P., Reis M.T., Rosado G.L., Pinheiro G.L., Mendes G.C., Fontes E.P. (2011). The NAC domain-containing protein, GmNAC6, is a downstream component of the ER stress- and osmotic stress-induced NRP-mediated cell-death signaling pathway. BMC Plant Biol..

[168] Reis P.A.B., Carpinetti P.A., Freitas P.P., Santos E.G., Camargos L.F., Oliveira I.H., Silva J.C.F., Carvalho H.H., Dal-Bianco M., Soares-Ramos J.R. (2016). Functional and regulatory conservation of the soybean ER stress-induced DCD/NRP-mediated cell death signaling in plants. BMC Plant Biol..

[169] Irsigler A.S., Costa M.D., Zhang P., Ab Reis P., Dewey R.E., Boston R.S., Fontes E.P. (2007). Expression profiling on soybean leaves reveals integration of ER- and osmotic-stress pathways. BMC Genom..

[170] Pimenta M.R., Alves R., Silva P.A., Mendes G.C., Brustolini B., Caetano D.N., Paulo J., Machado B., Jose O., Silva C.F. (2016). The Stress-Induced Soybean NAC Transcription Factor GmNAC81 Plays a Positive Role in Developmentally Programmed Leaf Senescence. Plant Cell Physiol..

[171] Mendes G.C., Reis P.A.B., Calil I.P., Carvalho H.H., Aragão F.J.L., Fontes E.P.B. (2013). GmNAC30 and GmNAC81 integrate the endoplasmic reticulum stress- and osmotic stress-induced cell death responses through a vacuolar processing enzyme. Proc. Natl. Acad. Sci. USA.

[172] Carvalho H.H., Silva P.A., Mendes G.C., Brustolini O.J., Pimenta M.R., Gouveia B.C., Valente M.A., Ramos H.J., Soares-Ramos J.R., Fontes E.P. (2014). The Endoplasmic Reticulum Binding Protein BiP Displays Dual Function in Modulating Cell Death Events. Plant Cell Physiol..

[173] Fernandez P., Di Rienzo J., Fernandez L., Hopp H.E., Paniego N., Heinz R.A. (2008). Transcriptomic identification of candidate genes involved in sunflower responses to chilling and salt stresses based on cDNA microarray analysis. BMC Plant Biol..

[174] Lavaud Y., Dosio G.A.A., González L.M., Aguirrezábal L.A.N., Izquierdo N.G., Andrade F.H. (2003). Intercepted Solar Radiation during Seed Filling Determines Sunflower Weight per Seed and Oil Concentration. Crop. Sci..

[175] Dosio G.A., Aguirreza´bal L.A., Pereyra V.R., Andrade F.H. (2000). Solar Radiation Intercepted during Seed Filling and Oil Production in Two Sunflower Hybrids. Crop. Sci..

[176] Hall A.J., Connor D.J., Whitfield D.M. (1989). Contribution of pre-anthesis assimilates to grain-filling in irrigated and water-stressed sunflower crops I. Estimates using labelled carbon. F. Crop. Res..

[177] Fernandez P., Soria M., Blesa D., DiRienzo J., Moschen S., Rivarola M., Clavijo B.J., Gonzalez S., Peluffo L., Príncipi D. (2012). Development, Characterization and Experimental Validation of a Cultivated Sunflower (*Helianthus annuus* L.) Gene Expression Oligonucleotide Microarray. PLoS ONE.

[178] Agüera E., Cabello P., De La Haba P. (2010). Induction of leaf senescence by low nitrogen nutrition in sunflower (*Helianthus annuus*) plants. Physiol. Plant..

[179] De La Mata L., Cabello P., De La Haba P., Agüera E. (2012). Growth under elevated atmospheric CO_2_ concentration accelerates leaf senescence in sunflower (*Helianthus annuus* L.) plants. J. Plant Physiol..

[180] Manavella P.A., Arce A.L., Dezar C.A., Bitton F., Renou J.-P., Crespi M., Chan R.L. (2006). Cross-talk between ethylene and drought signalling pathways is mediated by the sunflower Hahb-4 transcription factor. Plant J..

[181] Olsen A.N., Ernst H.A., Leggio L.L., Skriver K. (2005). NAC transcription factors: Structurally distinct, functionally diverse. Trends Plant Sci..

[182] Page T., Harrison E., Breeze E., Lim P.O., Gil Nam H., Lin J.F., Wu S.H., Swidzinski J., Ishizaki K., Leaver C.J. (2005). Comparative transcriptome analysis reveals significant differences in gene expression and signalling pathways between developmental and dark/starvation-induced senescence in Arabidopsis. Plant J..

[183] Gialdi A.L., Moschen S., Villán C., Fernández M.L., Maldonado S., Paniego N., Heinz R., Fernandez P., López A. (2016). Identification and characterization of contrasting sunflower genotypes to early leaf senescence process combining molecular and physiological studies (*Helianthus annuus* L.). Plant Sci..

[184] Dong H., Li W.J., Tang W., Zhang D.M. (2010). Research Progress in Physiological Premature Senescence in Cotton. Cotton Sci..

[185] Liu W., Zhang W., Zheng N., Zhai W., Qi F. (2018). Study of Cotton Leaf Senescence Induced by Alternaria Alternata Infection. Advanced Structural Safety Studies.

[186] Lin M., Pang C., Fan S., Song M., Wei H., Yu S. (2015). Global analysis of the *Gossypium hirsutum* L. Transcriptome during leaf senescence by RNA-Seq. BMC Plant Biol..

[187] Zhao F., Ma J., Li L., Fan S., Guo Y., Song M., Wei H., Pang C., Yu S. (2016). GhNAC12, a neutral candidate gene, leads to early aging in cotton (*Gossypium hirsutum* L). Gene.

[188] Zhong R., Demura T., Ye Z.H. (2006). SND1, a NAC Domain Transcription Factor, Is a Key Regulator of Secondary Wall Synthesis in Fibers of Arabidopsis. Plant Cell Online.

[189] Fan K., Bibi N., Gan S., Li F., Yuan S., Ni M., Wang M., Shen H., Wang X. (2015). A novel NAP member GhNAP is involved in leaf senescence in *Gossypium hirsutum*. J. Exp. Bot..

[190] Elasad M., Ondati E., Wei H., Wang H., Su J., Fan S., Pang C., Yu S. (2018). Functional analysis of nine cotton genes related to leaf senescence in *Gossypium hirsutum* L.. Physiol. Mol. Biol. Plants.

[191] Gu L., Dou L., Guo Y., Wang H., Li L., Wang C., Ma L., Wei H., Yu S. (2019). The WRKY transcription factor GhWRKY27 coordinates the senescence regulatory pathway in upland cotton (*Gossypium hirsutum* L.). BMC Plant Biol..

[192] Christ B., Süssenbacher I., Moser S., Bichsel N., Egert A., Müller T., Kräutler B., Hörtensteiner S. (2013). Cytochrome P450 CYP89A9 Is Involved in the Formation of Major Chlorophyll Catabolites during Leaf Senescence in Arabidopsis. Plant Cell.

[193] Diago M.P., Correa C., Millan B., Barreiro P., Valero C., Tardáguila J. (2012). Grapevine Yield and Leaf Area Estimation Using Supervised Classification Methodology on RGB Images Taken under Field Conditions. Sensors.

[194] Jaillon O., Aury J.M., Noel B., Policriti A., Clepet C., Casagrande A., Choisne N., Aubourg S., Vitulo N., Jubin C. (2007). French-Italian Public Consortium for Grapevine Genome Characterization The grapevine genome sequence suggests ancestral hexaploidization in major angiosperm phyla. Nature.

[195] Le Hénanff G., Profizi C., Courteaux B., Rabenoelina F., Gérard C., Clément C., Baillieul F., Cordelier S., Dhondt-Cordelier S. (2013). Grapevine NAC1 transcription factor as a convergent node in developmental processes, abiotic stresses, and necrotrophic/biotrophic pathogen tolerance. J. Exp. Bot..

[196] Zhu Z., Li G., Yan C., Liu L., Zhang Q., Han Z., Li B. (2019). DRL1, Encoding A NAC Transcription Factor, Is Involved in Leaf Senescence in Grapevine. Int. J. Mol. Sci..

[197] Katsumoto Y., Fukuchi-Mizutani M., Fukui Y., Brugliera F., Holton T.A., Karan M., Nakamura N., Yonekura-Sakakibara K., Togami J., Pigeaire A. (2007). Engineering of the Rose Flavonoid Biosynthetic Pathway Successfully Generated Blue-Hued Flowers Accumulating Delphinidin. Plant Cell Physiol..

[198] Nakatsuka T., Haruta K.S., Pitaksutheepong C., Abe Y., Kakizaki Y., Yamamoto K., Shimada N., Yamamura S., Nishihara M. (2008). Identification and Characterization of R2R3-MYB and bHLH Transcription Factors Regulating Anthocyanin Biosynthesis in Gentian Flowers. Plant Cell Physiol..

[199] Shibuya K., Shimizu K., Niki T., Ichimura K. (2014). Identification of a NAC transcription factor, EPHEMERAL1, that controls petal senescence in Japanese morning glory. Plant J..

[200] De La Torre M.C.P., Fernandez P., Greppi J.A., Coviella M.A., Fernández M.N., Astigueta F., Mata D.A., Trupkin S.A. (2018). Transformation of Mecardonia (*Plantaginaceae*) with wild-type Agrobacterium rhizogenes efficiently improves compact growth, branching and flower related ornamental traits. Sci. Hortic..

[201] Trupkin S.A., Astigueta F.H., Baigorria A.H., García M.N., Delfosse V.C., González S.A., de la Torre M.C., Moschen S., Lía V.V., Fernández P. (2019). Identification and expression analysis of NAC transcription factors potentially involved in leaf and petal senescence in Petunia hybrida. Plant Sci..

[202] Agarwal P., Kapoor S., Tyagi A.K. (2011). Transcription factors regulating the progression of monocot and dicot seed development. BioEssays.

[203] Ooka H., Satoh K., Doi K., Nagata T., Otomo Y., Murakami K., Matsubara K., Osato N., Kawai J., Carninci P. (2003). Comprehensive analysis of NAC family genes in *Oryza sativa* and *Arabidopsis thaliana*. Curr. Neuropharmacol..

[204] Tranbarger T.J., Fooyontphanich K., Roongsattham P., Pizot M., Collin M., Jantasuriyarat C., Suraninpong P., Tragoonrung S., Dussert S., Verdeil J.L. (2017). Transcriptome Analysis of Cell Wall and NAC Domain Transcription Factor Genes during Elaeis guineensis Fruit Ripening: Evidence for Widespread Conservation within Monocot and Eudicot Lineages. Front. Plant Sci..

[205] Pereira-Santana A., Alcaraz L.D., Castaño E., Sánchez-Calderón L., Sánchez-Teyer F., Rodriguez-Zapata L. (2015). Comparative Genomics of NAC Transcriptional Factors in Angiosperms: Implications for the Adaptation and Diversification of Flowering Plants. PLoS ONE.

[206] Jin X., Ren J., Nevo E., Yin X., Sun D., Peng J. (2017). Divergent Evolutionary Patterns of NAC Transcription Factors Are Associated with Diversification and Gene Duplications in Angiosperm. Front. Plant Sci..

[207] Khan M., Rozhon W., Poppenberger B. (2013). The role of hormones in the aging of plants—A mini-review. Gerontology.

[208] Vandenbussche M., Chambrier P., Rodrigues Bento S., Morel P. (2016). Petunia, Your Next Supermodel?. Front. Plant Sci..

